# Black-Scholes Theory and Diffusion Processes on the Cotangent Bundle of the Affine Group

**DOI:** 10.3390/e22040455

**Published:** 2020-04-17

**Authors:** Amitesh S. Jayaraman, Domenico Campolo, Gregory S. Chirikjian

**Affiliations:** 1 Department of Mechanical Engineering, National University of Singapore, Singapore 117575, Singapore; amitesh.sivaraman@u.nus.edu; 2 School of Mechanical and Aerospace Engineering, Nanyang Technological University, Singapore 639798, Singapore; d.campolo@ntu.edu.sg; 3 Department of Mechanical Engineering, Johns Hopkins University, Baltimore, MD 21218, USA

**Keywords:** Black-Scholes, affine, unimodular, cotangent bundle, Lie groups, diffusion

## Abstract

The Black-Scholes partial differential equation (PDE) from mathematical finance has been analysed extensively and it is well known that the equation can be reduced to a heat equation on Euclidean space by a logarithmic transformation of variables. However, an alternative interpretation is proposed in this paper by reframing the PDE as evolving on a Lie group. This equation can be transformed into a diffusion process and solved using mean and covariance propagation techniques developed previously in the context of solving Fokker–Planck equations on Lie groups. An extension of the Black-Scholes theory with coupled asset dynamics produces a diffusion equation on the affine group, which is not a unimodular group. In this paper, we show that the cotangent bundle of a Lie group endowed with a semidirect product group operation, constructed in this paper for the case of groups with trivial centers, is always unimodular and considering PDEs as diffusion processes on the unimodular cotangent bundle group allows a direct application of previously developed mean and covariance propagation techniques, thereby offering an alternative means of solution of the PDEs. Ultimately these results, provided here in the context of PDEs in mathematical finance may be applied to PDEs arising in a variety of different fields and inform new methods of solution.

## 1. Introduction

The Nobel Prize-winning Black-Scholes Equation [1] is arguably the most well-known partial differential equation in mathematical finance. The equation rests on a parsimonious option pricing model that has been fairly successful in informing banks and portfolio managers of the construction of risk-free hedges. Additionally, the model has since provided the framework for the import of a variety of tools, such as the theory of stochastic processes, from physics and mathematics. In this paper, we offer a new Lie group-theoretic interpretation of the Black-Scholes equation by reformulating the original equation and extensions of it as diffusion processes on Lie groups.

Group-theoretic approaches have been used extensively in the analysis of symmetries of partial differential equations (PDEs) in mathematical finance [2,3,4]. One of the central questions there is to identify the group of transformations of variables that can be applied to the equations that preserve the structure of the equations while reducing it to a simpler form for analysis and solution. For instance, the one-asset [2] and in general, the multi-asset Black Scholes equation [5] can be reduced to a heat equation through a logarithmic transformation of variables.

In this paper, instead of analysing the symmetry properties of PDEs, we reformulate the PDE as a diffusion equation on the Lie group. Functions of real parameters are upgraded to functions that take group elements as their arguments. That is, we start with a linear (parabolic) PDE of the form,

(1) Lf(q,t)=0,

where *L* is the linear partial differential operator and q is a vector of coordinates that may be chosen based on how the differential equation is derived. In the case of the Black-Scholes equation and related asset models considered in this paper, *L* and q are originally defined in an *N*-dimensional Euclidean space. By matching the derivatives in *L* to Lie derivatives of *correctly* chosen groups, one can rewrite (1) as the following linear PDE over the Lie group *G*,

(2) $$\tilde{L}\tilde{f}\left(g\left(q\right),t\right)=0,$$

where q parameterizes the group element g∈G and $\tilde{f}\left(g\left(q\right),t\right)=f\left(q,t\right)$. The operator $\tilde{L}$ is now a differential operator consisting of Lie directional derivatives. Such differential equations arise in bevel-tip needle steering [6,7], error propagation [8,9], DNA statistical mechanics [10], multi-robot localization [11], stochastic kinematic cart models in SLAM [12] and in image contour completion and enhancement [13,14,15].

The benefit of reframing differential equations this way would be that variable-coefficient PDEs of *N* variables in $R^{N}$ may reduce to constant-coefficient PDEs on the Lie group *G* of dimension of at least *N*, which can be analysed using techniques developed in [6,7,8,9,10,11,12]. These applications motivated the development of mean and covariance propagation techniques to approximate the solution of diffusion equations in the special Euclidean group SE(N) and more generally, in unimodular groups [16,17]. Other numerical techniques to solve differential equations on Lie groups, through a generalisation of Euler and Runge-Kutta schemes have been developed in [18,19,20].

In this paper, we extend the regime of applicability of mean and covariance propagation techniques by first considering the groups $GL^{+}\left(1\right)$ and $GL^{+}\left(1\right)\times GL^{+}\left(1\right)$ that arise in the one-asset and two-asset Black-Scholes equations, respectively, and then the affine group, $Aff^{+}\left(1\right)$ that arises in a coupled-asset dynamics extension of the Black-Scholes theory. The application of mean and covariance propagation to $Aff^{+}\left(1\right)$ becomes the main subject of the paper. There exist other methods to approximately solve PDEs in mathematical finance, such as finite difference methods [21,22], finite element methods [23] and the Adomian decomposition method [24,25]. In this paper, we also make a comparison between the mean and covariance propagation technique and a standard finite difference scheme used to solve the governing equations.

The Lie group $Aff^{+}\left(1\right)$ is not unimodular, thereby precluding the direct application of the theory of mean and covariance propagation used in the solution of diffusion equations on the group. However, we show that the cotangent bundle group of a Lie group (with a trivial center) is unimodular. Thus, we analyse diffusion processes on the affine group by matching the diffusion on the affine group with a degenerate diffusion on the cotangent bundle to which mean and covariance propagation techniques can be applied.

This paper considers three types of asset dynamics models: one-asset, two-asset and a coupled asset model. Each of these models give rise to a PDE describing the evolution of the option price as a function of the asset prices, and are introduced in Section 2. These PDEs in Euclidean space are reframed as diffusion processes on Lie groups in Section 3. The theory of mean and covariance propagation in the solution of diffusion processes on Lie groups is reviewed in Section 4. The one-asset and two-asset Black-Scholes models, which can be trivially solved using the logarithmic transformation of variables, are used as examples to illustrate the techniques, and thereby sets the ground for the non-trivial case of a coupled asset model. Since the coupled asset model leads to a diffusion equation over a non-unimodular group, Section 5 proves that a related structure, the cotangent bundle group of a Lie group with trivial center, is always unimodular. Section 6 describes the mean and covariance propagation over the unimodular cotangent bundle of the affine group, thereby solving the option price dynamics for the coupled asset model. Section 7 provides some numerical results for the mean and covariance propagated solution in comparison with finite difference methods. Finally, Section 8 seeks to demonstrate backward compatibility and completes the analysis by applying mean and covariance propagation on the cotangent bundle group to the solution of the one-asset Black-Scholes equation.

## 2. Asset Dynamics Models

In this section, we review the derivation of the well-known one-asset Black-Scholes equation, the two-asset Black-Scholes equation that evolve with correlated Wiener process, and follow a similar derivation to develop a coupled asset model.

### 2.1. One-Asset Black-Scholes Equation

The one-asset Black-Scholes equation is derived from the following Itô stochastic differential equation [26] governing the dynamics of an asset value *a*,

(3) da/a=μdt+σdW,

where dW is the increment of a Wiener process, corresponding to random draws from a Gaussian probability distribution [27,28]. The increment of the Wiener process satisfies the following relations:

(4) $$\begin{array}{cc} \langle dW\rangle & =0\\ \langle dW^{2}\rangle & =dt, \end{array}$$

and in general for an *N*-dimensional Wiener increment dW we have,

(5) $$\begin{array}{cc} \langle dW\rangle & =0\\ \langle dW_{i}dW_{j}\rangle & =dt\delta_{ij}, \end{array}$$

where in both cases 〈⋯〉 denotes the expected value with respect to the underlying probability distribution function and $\delta_{ij}$ is the Kronecker-delta ($\delta_{ij}=1$ if and only if i=j, otherwise $\delta_{ij}=0$). The μ and σ in Equation (3) respectively describe a drift and volatility for the asset price evolution. The volatility is defined such that $\sigma^{2}a^{2}$ is the variance of the random price fluctuation. Equation (3) is a stochastic differential equation whose solution is a geometric Brownian motion. We emphasise that the equation in (3) is to be interpreted as an Itô stochastic differential equation. Stratonovich stochastic differential equations also appear in this paper (see Equation (110) for instance) and will be distinguished from Itô equations with a Ⓢ, i.e., for a stochastically varying quantity *x*, a general one-dimensional Stratonovich stochastic differential equation with Wiener noise would be,

dx=a(x,t)dt+b(x,t)ⓈdW,

for a drift a(x,t) and diffusion coefficient b(x,t). Nevertheless the properties of dW in (4) and of dW in (5) hold for both Itô and Stratonovich equations.

The option price V=V(a,t) is a function of time *t* and the underlying asset price *a*. Using Itô’s Lemma [29], we have for dV correct to order dt,

(6) $$\begin{array}{cc} dV & =\frac{\partial V}{\partial t}dt+\frac{\partial V}{\partial a}da+\frac{1}{2}\frac{\partial^{2}V}{\partial a^{2}}da^{2}\\ \; & =\left(\frac{\partial V}{\partial t}+\mu a\frac{\partial V}{\partial a}+\frac{\sigma^{2}}{2}a^{2}\frac{\partial^{2}V}{\partial a^{2}}\right)dt+\sigma a\frac{\partial V}{\partial a}dW. \end{array}$$

In the Black-Scholes model, there also exists another asset, the bond price, which evolves deterministically (for a non-stochastic interest rate) as dB=rBdt where *r* is the risk-free interest rate [26]. A portfolio Π is constructed as a linear combination of the two assets and can be written as,

(7) Π=Δa+βB.

This form of Π is due to the fact that the portfolio is assumed to be self-financing with no external money flows [30]. Banks choose Δ in order to obtain a zero-risk portfolio [31], i.e., so that d(V+Π) would have no Wiener noise terms. Assets without stochasticity would evolve at the risk-free interest rate *r* and therefore we obtain,

(8) d(V+Π)=r(V+Π)dt,

and using dΠ=ΔdV+βdB, a hedge of the form Δ=−a∂V/∂a would cancel out the (σa∂V/∂a)dW term in (6) and therefore make the dynamics of V+Π risk-free. Substituting this hedge into (8) and using the form of dV in (6) we obtain the one-asset Black-Scholes equation as,

(9) $$\frac{\partial V}{\partial t}+\frac{1}{2}\sigma^{2}a^{2}\frac{\partial^{2}V}{\partial a^{2}}+ra\frac{\partial V}{\partial a}-rV=0.$$

The parameters β and μ do not feature in this equation.

### 2.2. Two-Asset Black-Scholes Equation

In the two-asset model, one has asset values *a* and *b* that evolve with correlated Wiener processes—this is a specific case of the multi-asset model in [5]. That is,

(10) $$\begin{array}{cc} da/a & =\mu_{1}dt+\sigma_{1}dW_{1}\\ db/b & =\mu_{2}dt+\sigma_{2}dW_{2}, \end{array}$$

where $\langle dW_{1}dW_{2}\rangle =\rho dt$ and 〈⋯〉 represents the expectation. In the two-asset problem, the option price is V=V(a,b,t). Applying Itô’s Lemma to dV now gives:

(11) $$dV=\left(\frac{\partial V}{\partial t}+\mu_{1}a\frac{\partial V}{\partial a}+\mu_{2}b\frac{\partial V}{\partial b}+\rho \sigma_{1}\sigma_{2}ab\frac{\partial^{2}V}{\partial a\partial b}+\frac{\sigma_{1}^{2}}{2}a^{2}\frac{\partial^{2}V}{\partial a^{2}}+\frac{\sigma_{2}^{2}}{2}b^{2}\frac{\partial^{2}V}{\partial b^{2}}\right)dt+\sigma_{1}a\frac{\partial V}{\partial a}dW_{1}+\sigma_{2}b\frac{\partial V}{\partial b}dW_{2}.$$

The portfolio is now $\Pi =\Delta_{1}a+\Delta_{2}b+\beta B$ where $\Delta_{1}=-a\partial V/\partial a$ and $\Delta_{2}=-b\partial V/\partial b$; $\Delta_{1}$ and $\Delta_{2}$ are both chosen such that V+Π experiences risk-free dynamics—very similar to the procedure in the derivation of the one-asset Black-Scholes equation in the previous section. The two-asset Black-Scholes equation [32] will then be,

(12) $$\frac{\partial V}{\partial t}+ra\frac{\partial V}{\partial a}+rb\frac{\partial V}{\partial b}+\frac{\sigma_{1}^{2}}{2}a^{2}\frac{\partial^{2}V}{\partial a^{2}}+\rho \sigma_{1}\sigma_{2}ab\frac{\partial^{2}V}{\partial a\partial b}+\frac{\sigma_{2}^{2}}{2}b^{2}\frac{\partial^{2}V}{\partial b^{2}}-rV=0,$$

where *r* is the risk-free interest rate. Like the one-asset Black-Scholes equation, $\mu_{1},\mu_{2}$ and β do not feature in the two-asset equation.

### 2.3. Option Price Evolution with Coupled Assets

The evolution equations of the two assets *a* and *b* in the two-asset Black-Scholes model (10) featured correlated Wiener processes. In this section, we imagine a different form of coupling of the form:

(13) $$\begin{array}{cc} da/a & =\mu_{1}dt+\sigma_{1}dW\\ db/a & =\mu_{2}dt+\sigma_{2}dW. \end{array}$$

The evolution of the value of asset *a* is independent of the evolution of *b*; *a* is therefore the ‘leading’ asset value. On the other hand, the drift and variance in the stochastic differential equation for db are dependent on the current values of the leading asset. Hence *b* is the ‘trailing’ asset value. One may imagine this form of dependency when *a* represents a raw material and *b* represents a finished good that makes use of this raw material.

Both da and db are forced by the same Wiener process dW; this implies that $\sigma_{2}/\sigma_{1}=\left(\mu_{2}-r\right)/\left(\mu_{1}-r\right)$ for the risk-free interest rate *r*. This can be derived by using the risk-neutral measure with the knowledge that all assets evolve with a risk-free interest rate of *r* in this measure [31]. The option price is V=V(a,b,t) and applying Itô’s Lemma to dV now gives,

(14) $$dV=\left(\frac{\partial V}{\partial t}+\mu_{1}a\frac{\partial V}{\partial a}+\mu_{2}a\frac{\partial V}{\partial b}+\sigma_{1}\sigma_{2}a^{2}\frac{\partial^{2}V}{\partial a\partial b}+\frac{\sigma_{1}^{2}}{2}a^{2}\frac{\partial^{2}V}{\partial a^{2}}+\frac{\sigma_{2}^{2}}{2}a^{2}\frac{\partial^{2}V}{\partial b^{2}}\right)dt+\left(\sigma_{1}a\frac{\partial V}{\partial a}+\sigma_{2}a\frac{\partial V}{\partial b}\right)dW.$$

We now assume a general hedge of the form $\Pi =\Delta_{1}a+\Delta_{2}b+\beta B$. Since there is only one Wiener process dW, removing uncertainty would not be a sufficient condition to determine both $\Delta_{1}$ and $\Delta_{2}$. Thus, for simplicity, we let $\Delta_{2}=0$ which leads to the relationship $\Delta_{1}=-\partial V/\partial a-\left(\sigma_{2}/\sigma_{1}\right)\partial V/\partial b$. The governing partial differential equation for V(a,b,t) with two coupled assets would be,

(15) $$\frac{\partial V}{\partial t}+ra\frac{\partial V}{\partial a}+\left[\left(\frac{\mu_{2}}{\mu_{1}}-\frac{\sigma_{2}}{\sigma_{1}}\right)\mu_{1}+\frac{\sigma_{2}}{\sigma_{1}}r\right]a\frac{\partial V}{\partial b}+\frac{\sigma_{1}^{2}}{2}a^{2}\frac{\partial^{2}V}{\partial a^{2}}+\sigma_{1}\sigma_{2}a^{2}\frac{\partial^{2}V}{\partial a\partial b}+\frac{\sigma_{2}^{2}}{2}a^{2}\frac{\partial^{2}V}{\partial b^{2}}-rV=0,$$

where the leading asset/trailing asset coupling between the variables breaks the symmetry between the assets *a* and *b* that existed in (12).

## 3. Reframing Partial Differential Equations as Diffusion Processes on Lie Groups

The governing PDEs for the three types of asset dynamics models in (9), (12) and (15) can be re-expressed in terms of Lie derivatives of $GL{\left(1\right)}^{+}$, $GL{\left(1\right)}^{+}\times GL{\left(1\right)}^{+}$ and $Aff^{+}\left(1\right)$, respectively, by matching the group parameters to the asset variables. A review of the concept of Lie derivatives is presented in Section 3.1.

### 3.1. Preliminary Definitions

Let *G* be an *N*-dimensional matrix Lie group with Lie algebra G. Then, let an element g∈G be parameterized as g=g(q) where $q\in R^{N}$, using the notation in [29,33]. The ‘right’ Jacobian $J_{R}\left(g\left(q\right)\right)$ of the group is defined [34] as the following matrix,

(16) $$\left[J_{R}\left(g\left(q\right)\right)\right]=\left[{\left(g^{-1}\frac{\partial g}{\partial q_{1}}\right)}^{\vee },\cdots ,{\left(g^{-1}\frac{\partial g}{\partial q_{N}}\right)}^{\vee }\right],$$

and the ‘left’ Jacobian $J_{L}\left(g\left(q\right)\right)$ is the matrix,

(17) $$\left[J_{L}\left(g\left(q\right)\right)\right]=\left[{\left(\frac{\partial g}{\partial q_{1}}g^{-1}\right)}^{\vee },\cdots ,{\left(\frac{\partial g}{\partial q_{N}}g^{-1}\right)}^{\vee }\right],$$

where the square brackets reinforce that we are dealing with a matrix. This is not to be confused with the ‘right’ and ‘left’ Jacobian determinants that arise in the volume forms of the group, which would be the determinants of the matrices in (16) and (17), respectively. Note that the ‘right’ Jacobian has the $g^{-1}$ appearing on the right whereas the ‘left’ Jacobian has the term on the left. However, the ‘right’ Jacobian is left invariant, i.e., $J_{R}\left(h\circ g\left(q\right)\right)=J_{R}\left(g\left(q\right)\right)$ and the ‘left’ Jacobian is right invariant, i.e., $J_{L}\left(g\left(q\right)\circ h\right)=J_{L}\left(g\left(q\right)\right)$, assuming that q parameterizes the whole group *G* and that these shifts are permitted in the function domain. Finally, the ∨ operator is defined as a bijection mapping G to $R^{N}$, and vectorizes the matrix element in G. The inverse of the ∨ operator is a ∧ that maps $R^{N}$ to G.

We make an additional remark regarding the parameterization of the group with q. This is to say that the whole group (except for a set of measure zero) is parameterized by one coordinate chart. For instance, for groups such as SO(3) or SE(3) where one may use Euler angles to parameterize the rotations, there exists a set of measure zero corresponding to the set of Euler angles where the Jacobian matrices for the parameterization becomes singular. For (α,β,γ) denoting the ZXZ or ZYZ Euler angles, singularities occur at β=0 and β=π. Additionally, since a rotation by (α,β,γ) describes the same rotation as that by (α+2π,β,γ+2π), the open coordinate chart will be (α,β,γ)∈(−π,π)×(0,π)×(−π,π), which has a one-to-one correspondence with a subset of rotations that excludes the rotations at β=0 and π, and the rotations at α=π and γ=π. The closure of this coordinate chart will however establish a many-to-one map with the group and parameterize all group elements. A similar issue exists in the case of using the Iwasawa decomposition to parameterize SL(2,R) with (θ,λ,ξ) where θ parameterizes the 2D rotation, and (λ,ξ) parameterize the 2×2 upper triangular matrix. Here, the coordinate chart would be $\left(\theta ,\lambda ,\xi \right)\in \left(-\pi ,\pi \right)\times R^{+}\times R$; yet again, the closure of this chart will establish a many-to-one map with SL(2,R) and parameterize all group elements. For other cases, such as using a vector drawn from $R^{N^{2}}$ to parameterize GL(N) as well as for the affine group $Aff^{+}\left(1\right)$ and the cotangent bundle group of $Aff^{+}\left(1\right)$ considered in this paper, the coordinate chart used parameterizes the entire group.

The operator of the adjoint representation of group *G* at g∈G is given by Ad(g) where $Ad\left(g\right)X=gXg^{-1}$ for any X∈G. When expressed as a matrix, we denote the operator as [Ad(g)]. If $E_{i}$ for i=1,⋯,N forms an *N*-dimensional orthonormal basis for G, [Ad(g)] is given as,

(18) $$\left[Ad\left(g\right)\right]=\left[{\left(gE_{1}g^{-1}\right)}^{\vee },\cdots ,{\left(gE_{N}g^{-1}\right)}^{\vee }\right].$$

Orthonormality of the Lie algebra basis is defined here with respect to an inner product of the form $\left(E_{i},E_{j}\right)=e_{i}^{T}e_{j}$ where $E_{i}^{\vee }=e_{i}$, effectively fixing a metric for *G*. Using $\left[E_{i},E_{j}\right]=E_{i}E_{j}-E_{j}E_{i}$ to define the Lie bracket, we can also represent the “little ad” operator, where ad(X)Y=[X,Y] for X,Y∈G as,

(19) $$\left[ad\left(X\right)\right]=\left[{\left[X,E_{1}\right]}^{\vee },\cdots ,{\left[X,E_{N}\right]}^{\vee }\right],$$

where X∈G and Ad(exp(X))=exp(ad(X)).

For a differentiable function on the group f:G→R, one can construct the right and left Lie directional derivatives as,

(20) $$E_{i}^{R}f\left(g\right)\;\dot{=}\;{\left.\frac{d}{dt}f\left(g\circ \exp\left(tE_{i}\right)\right)\right|}_{t=0}\;\;\mathrm{and}\;\;E_{i}^{L}f\left(g\right)\;\dot{=}\;{\left.\frac{d}{dt}f\left(\exp\left(-tE_{i}\right)\circ g\right)\right|}_{t=0}.$$

In parametric form,

(21) $$E^{R}f\left(g\left(q\right)\right)={\left[{\tilde{J}}_{R}\left(q\right)\right]}^{-T}\nabla_{q}\tilde{f}\left(q\right)\;\;\mathrm{and}\;\;E^{L}f\left(g\left(q\right)\right)={\left[{\tilde{J}}_{L}\left(q\right)\right]}^{-T}\nabla_{q}\tilde{f}\left(q\right),$$

where $\tilde{f}\left(q\right)=f\left(g\left(q\right)\right)$, ${\tilde{J}}_{R,L}\left(q\right)=J_{R,L}\left(g\left(q\right)\right)$ and $\nabla_{q}={\left[\partial /\partial q_{1},\cdots ,\partial /\partial q_{N}\right]}^{T}$ is the gradient operator for $R^{N}$. Here, ‘−T’ represents the inverse transpose operation, i.e., the inverse of the transpose of the matrix. The Lie directional derivative operators are $E^{R}={\left[E_{1}^{R},\cdots ,E_{N}^{R}\right]}^{T}$ and $E^{L}={\left[E_{1}^{L},\cdots ,E_{N}^{L}\right]}^{T}$. In the sequel, we drop the tildes, as it will be clear from the arguments whether f(g(q)) or $\tilde{f}\left(q\right)$ is considered, and likewise for the Jacobians.

### 3.2. One-Asset Black-Scholes as a Diffusion on $Gl^{+}\left(1\right)$

The set of positive real numbers equipped with the multiplication operation forms a commutative (Abelian) group. This is a subgroup of the general linear group of one dimension and is represented by $GL^{+}\left(1\right)$. The basis of the Lie algebra is the number 1 and for a group element $a\in GL^{+}\left(1\right)$, the corresponding element in the Lie algebra is loga. Using (21) for a differentiable function $f:GL^{+}\left(1\right)\to R$, we obtain,

(22) $$Ef\left(a\right)=a\frac{\partial f}{\partial a},$$

where both left and right Lie derivatives yield the same result since the group is Abelian.

Using this relationship, we can rewrite the one-asset Black-Scholes Equation (9) as,

(23) $$\frac{\partial V}{\partial t}+\frac{\sigma^{2}}{2}E^{2}V+\left(r-\frac{\sigma^{2}}{2}\right)EV-rV=0,$$

using the shorthand $E^{2}V=E\left(EV\right)$. The variable-coefficient Black-Scholes equation has thus been transformed to a constant-coefficient equation on $GL^{+}\left(1\right)$. Additionally, using time reversal $t^{\prime }=-t$ to convert the backward parabolic equation to a forward parabolic equation and setting $V\left(a,t^{\prime }\right)=u\left(a,t^{\prime }\right)e^{-rt^{\prime }}$ we have,

(24) $$\frac{\partial u}{\partial t^{\prime }}=\left(r-\frac{\sigma^{2}}{2}\right)Eu+\frac{\sigma^{2}}{2}E^{2}u,$$

which is a diffusion equation with drift $r-\sigma^{2}/2$ and diffusivity $\sigma^{2}$. Here, $u=u(a,t^{\prime })$ is interpreted as a function over $GL^{+}\left(1\right)\times R_{\geq 0}$. Henceforth the term initial condition will be with respect to $t^{\prime }$, which due to time reversal, refers to a final condition with respect to *t*.

We note that (9) by itself is not a diffusion process. Instead, we obtain a diffusion equation in (24) only after the time reversal $t^{\prime }=-t$ and making the exponential transformation $V=u\left(a,t^{\prime }\right)e^{-rt^{\prime }}$. Therefore, solving for $u(a,t^{\prime })$ indirectly solves for $V(a,t^{\prime })$ and solves the Black-Scholes equations. In subsequent sections, we apply similar transformations, while noting that solutions of such diffusion equations for *u* indirectly provides a solution for *V*.

### 3.3. Two-Asset Black-Scholes as a Diffusion on $Gl^{+}\left(1\right)\times Gl^{+}\left(1\right)$

An element $g\in GL^{+}\left(1\right)\times GL^{+}\left(1\right)$ parameterized as g=g(a,b) can be represented by a diagonal matrix as,

(25) $$g=\left(\begin{array}{ccc} a & \; & 0\\ 0 & \; & b \end{array}\right).$$

The Lie algebra of $GL^{+}\left(1\right)\times GL^{+}\left(1\right)$ can be represented by the orthonormal basis,

(26) $$E_{1}=\left(\begin{array}{ccc} 1 & \; & 0\\ 0 & \; & 0 \end{array}\right)\;\;\mathrm{and}\;\;E_{2}=\left(\begin{array}{ccc} 0 & \; & 0\\ 0 & \; & 1 \end{array}\right).$$

The group is Abelian and much like $GL^{+}\left(1\right)$, the left and right Lie derivatives coincide for a differentiable function $f:GL^{+}\left(1\right)\times GL^{+}\left(1\right)\to R$ as,

(27) $$\left(\begin{array}{c} E_{1}f\left(g\right)\\ E_{2}f\left(g\right) \end{array}\right)=\left(\begin{array}{c} a\;\partial \tilde{f}/\partial a\\ b\;\partial \tilde{f}/\partial b \end{array}\right),$$

where $f\left(g\left(a,b\right)\right)=\tilde{f}\left(a,b\right)$. This allows us to write the two-asset Black-Scholes Equation (12) in terms of Lie derivatives as,

(28) $$\frac{\partial u}{\partial t^{\prime }}=\left(r-\frac{\sigma_{1}^{2}}{2}\right)E_{1}u+\left(r-\frac{\sigma_{2}^{2}}{2}\right)E_{2}u+\frac{1}{2}\left(\sigma_{1}^{2}E_{1}^{2}+2\rho \sigma_{1}\sigma_{2}E_{1}E_{2}+\sigma_{2}^{2}E_{2}^{2}\right)u,$$

where $t^{\prime }=-t$ and $V\left(a,b,t^{\prime }\right)=u\left(g\left(a,b\right),t^{\prime }\right)e^{-rt^{\prime }}$. Hence, the solution of (28) allows one to construct the solution to the two-asset Black-Scholes Equation (12).

### 3.4. Option Price Evolution with Coupled Assets as a Diffusion on $Aff^{+}\left(1\right)$

The affine group of the positive real line consists of all $\left(a,b\right)\in R^{+}\times R$ that transforms the scalar x∈R to ax+b∈R that is, $Aff^{+}\left(1\right)=GL^{+}\left(1\right)⋉R$. An element $g\in Aff^{+}\left(1\right)$ can be expressed as,

(29) $$g=\left(\begin{array}{ccc} a & \; & b\\ 0 & \; & 1 \end{array}\right).$$

The group action is a matrix multiplication of *g* with $x={\left[x,1\right]}^{T}$ (where *x* is expressed in homogeneous coordinates as x). The Lie algebra of $Aff^{+}\left(1\right)$ is two dimensional and spanned by the orthonormal basis elements $E_{1}$ and $E_{2}$,

(30) $$E_{1}=\left(\begin{array}{ccc} 1 & \; & 0\\ 0 & \; & 0 \end{array}\right)\;\;\mathrm{and}\;\;E_{2}=\left(\begin{array}{ccc} 0 & \; & 1\\ 0 & \; & 0 \end{array}\right).$$

Since $\left(a,b\right)\in R^{+}\times R$, for $X=x_{1}E_{1}+x_{2}E_{2}$ in the Lie algebra of $Aff^{+}\left(1\right)$, $\left(x_{1},x_{2}\right)\in R^{2}$. Or equivalently for $a=e^{\alpha }$, we would have $\left(\alpha ,b\right)\in R^{2}$. Using (18), the adjoint representation of $Aff^{+}\left(1\right)$ would be,

(31) $$\left[Ad\left(g\right)\right]=\left(\begin{array}{ccc} 1 & \; & 0\\ -b & \; & a \end{array}\right).$$

An important feature of $Aff^{+}\left(1\right)$ is that the determinant of [Ad(g)] is *a*, which is generally not equal to 1. This implies that the group is not unimodular. For non-unimodular groups, there exist distinct left-invariant and right-invariant Haar measures [35,36]. Using (16) and (17), the left and right Jacobians $J_{L}$ and $J_{R}$ of $Aff^{+}\left(1\right)$ expressed as matrices are,

(32) $$\left[J_{L}\right]=\left(\begin{array}{ccc} 1/a & \; & 0\\ -b/a & \; & 1 \end{array}\right)\;\;\mathrm{and}\;\;\left[J_{R}\right]=\left(\begin{array}{ccc} 1/a & \; & 0\\ 0 & \; & 1/a \end{array}\right),$$

and since $\det\;J_{L}\neq \det\;J_{R}$ the Lie group is not unimodular.

The left and right Lie directional derivatives can be evaluated using (21) for a function $f:Aff^{+}\left(1\right)\to R$ as,

(33) $$\left(\begin{array}{c} E_{1}^{L}f\\ E_{2}^{L}f \end{array}\right)=\left(\begin{array}{c} a\;\partial \tilde{f}/\partial a+b\;\partial \tilde{f}/\partial b\\ \partial \tilde{f}/\partial b \end{array}\right)\;\;\mathrm{and}\;\;\left(\begin{array}{c} E_{1}^{R}f\\ E_{2}^{R}f \end{array}\right)=\left(\begin{array}{c} a\;\partial \tilde{f}/\partial a\\ a\;\partial \tilde{f}/\partial b \end{array}\right),$$

where $f\left(g\left(a,b\right)\right)=\tilde{f}\left(a,b\right)$. Therefore, we can now rewrite (15) in terms of Lie derivatives of $Aff^{+}\left(1\right)$ as

(34) $$\frac{\partial u}{\partial t^{\prime }}=\left(r-\frac{\sigma_{1}^{2}}{2}\right)E_{1}^{R}u+\left[\left(\frac{\mu_{2}}{\mu_{1}}-\frac{\sigma_{2}}{\sigma_{1}}\right)\mu_{1}+\frac{\sigma_{2}}{\sigma_{1}}r-\frac{\sigma_{1}\sigma_{2}}{2}\right]E_{2}^{R}u+\frac{1}{2}\left(\sigma_{1}^{2}{\left(E_{1}^{R}\right)}^{2}+\sigma_{1}\sigma_{2}\left(E_{2}^{R}E_{1}^{R}+E_{1}^{R}E_{2}^{R}\right)+\sigma_{2}^{2}{\left(E_{2}^{R}\right)}^{2}\right)u,$$

where $t^{\prime }=-t$ and $V\left(a,b,t^{\prime }\right)=u\left(g\left(a,b\right),t^{\prime }\right)e^{-rt^{\prime }}$. Hence, the solution of (34) allows one to construct the solution to the coupled asset model in Equation (15).

## 4. Mean and Covariance Propagation for Unimodular Lie Groups

Diffusion equations on unimodular Lie groups, such as (24) and (28) can be solved approximately using mean and covariance propagation techniques developed in [6,7,8,9,10,11,12,16]. This technique would not be applicable directly on the affine group in the solution of Equation (34) since the group is not unimodular; instead, the later sections will show how the technique can be modified by converting (34) to a diffusion over the cotangent bundle of the affine group, which is unimodular. This section will review the theory of covariance propagation for a general *N*-dimensional unimodular matrix Lie group *G* with Lie algebra G and apply the technique to solve (24) and (28).

Consider a general diffusion equation for u(g,t) on *G* in terms of right Lie derivatives as,

(35) $$\frac{\partial u}{\partial t}=-\sum_{i=1}^{N}m_{i}\left(t\right)E_{i}^{R}u+\frac{1}{2}\sum_{i=1}^{N}\sum_{j=1}^{N}D_{ij}\left(t\right)E_{i}^{R}E_{j}^{R}u,$$

where the drift vector $m\left(t\right)={\left[m_{1}\left(t\right),\cdots ,m_{N}\left(t\right)\right]}^{T}$ and diffusivity matrix D(t) are independent of *g* but can be time-dependent in general. Since the equation is a Fokker–Planck equation over a Lie group [17,33], one can interpret u(g,t) as a probability density (see Appendix B). This analogy also assumes that u(g,t) is square-integrable in *G*. Additionally, $\int_{G}u\left(g,0\right)dg=1$, which also holds true for all values of time. Here, dg is the Haar measure [35,36].

Given an initial condition $u_{0}=u\left(g,0\right)$, we aim to propagate the solution to the next time-step (t=δt) to obtain u(g,δt). In general, knowing u(g,t) we seek to obtain the solution at u(g,t+δt). The solution at t+δt can be obtained by a group convolution [33] from the solution at *t* as,

(36) $$\begin{array}{cc} u(g,t+\delta t) & =u\left(g,t\right)*u_{f}\left(g,\delta t\right)\\ \; & =\int_{G}u\left(h,t\right)u_{f}\left(h^{-1}\circ g,\delta t\right)\;dh, \end{array}$$

where $u_{f}\left(g,\delta t\right)$ is the fundamental solution/Green’s function that propagates the solution over δt. We recognise that over a sufficiently small δt the fundamental solution evolves in G. Since G can be bijectively mapped to the Euclidean space $R^{N}$, an approximate expression for $u_{f}\left(g,\delta t\right)$ would be a multivariate Gaussian that is a function of the *N*-dimensional q used to parameterize the group element g∈G. This Gaussian would evolve in $R^{N}$ with a drift of mδt. This drift can be injected on the group *G* by an evolution of the form $\mu \left(\delta t\right)=\exp\left(m^{\wedge }\delta t\right)$ where ∧ maps an element of $R^{N}$ to G [12,17]. The covariance of this Gaussian would be Dδt, which would equal the group-theoretic covariance Σ(δt) when restricted to small δt. The definitions of the group-theoretic mean, μ(t), and covariance, Σ(t), would be provided later in (39) and (40), respectively, but here it suffices to note that the group-theoretic mean and covariance match the mean and covariance defined in Euclidean space $R^{N}$ (see Appendix C) for small δt, thereby allowing us to construct $u_{f}\left(g,\delta t\right)$ in terms of the group parameters [17,33] as,

(37) $$u_{f}\left(g,\delta t\right)=\frac{1}{{\left(2\pi \right)}^{N/2}{\left|\det\;\Sigma \left(\delta t\right)\right|}^{1/2}}\exp\left(-\frac{1}{2}{\left[\log{\left(\mu {\left(\delta t\right)}^{-1}\circ g\right)}^{\vee }\right]}^{T}\Sigma^{-1}\left(\delta t\right)\left[\log{\left(\mu {\left(\delta t\right)}^{-1}\circ g\right)}^{\vee }\right]\right),$$

where the log maps the group element to G and ∨ maps the element of the Lie algebra to $R^{N}$. This form naturally extends to a Gaussian over the group in (47). If $u_{0}=u\left(g,0\right)=\delta_{G}\left(gg_{0}^{-1}\right)$, which is a group Dirac delta distribution centred about $g_{0}$, the distribution at δt remains tightly focused (the rigorous definition of such a distribution is provided in [9]) and $u\left(g,\delta t\right)=u_{f}\left(g,\delta t\right)$ from (36). The propagated solution would be,

(38) $$u\left(g,n\delta t\right)\approx u_{0}\left(g\right)*\underset{n\;\mathrm{times}}{\underset{︸}{u(g,\delta t)*u(g,\delta t)\cdots u(g,\delta t)}},$$

which is a specialized case of propagation of probability density using transition probabilities in a continuous-time Markovian process. We can define the group-theoretic mean μ(t) and covariance Σ(t) using the following equations [17,33]:

(39) $$\int_{G}\left[\log{\left(\mu {\left(t\right)}^{-1}\circ g\right)}^{\vee }\right]u\left(g,t\right)\;dg=0.$$

(40) $$\Sigma \left(t\right)\;\dot{=}\;\int_{G}\left[\log{\left(\mu {\left(t\right)}^{-1}\circ g\right)}^{\vee }\right]{\left[\log{\left(\mu {\left(t\right)}^{-1}\circ g\right)}^{\vee }\right]}^{T}u\left(g,t\right)\;dg,$$

where $\left[\log{\left(\mu {\left(t\right)}^{-1}\circ g\right)}^{\vee }\right]$ defines a distance from the mean. Note that $\int_{G}{\left[\log g\right]}^{\vee }u\left(g,t\right)dg$ is usually only approximately equal to $\log{\left(\mu \left(t\right)\right)}^{\vee }$ [8]. Similar definitions of mean and covariance are constructed in [37,38,39] and alternative definitions of an algebraic covariance have also been discussed in [17,40,41].

It is possible to obtain expressions for Σ(t) and μ(t) without explicitly solving for u(g,t) using the concepts of mean and covariance propagation developed in [6,7,8,9,10,11,12,16] by using the relation in (38). For X,Y,Z∈G, we have the Baker-Campbell-Hausdorff (BCH) formula:

$$e^{Z(X,Y)}=e^{X}e^{Y},$$

where [8,11],

(41) $$Z\left(X,Y\right)=X+Y+\frac{1}{2}\left[X,Y\right]+\frac{1}{12}(\left[X,\left[X,Y\right]+\left[Y,\left[Y,X\right]\right]\right)+\frac{1}{48}\left(\left[Y,\left[X,\left[Y,X\right]\right]\right]+\left[X,\left[Y,\left[Y,X\right]\right]\right]\right)\cdots .$$

Here, [X,Y]=XY−YX, is the Lie bracket in G. Since $x=\log{\left(e^{X}\right)}^{\vee }$ and making use of the fact that ${\left[X,Y\right]}^{\vee }=\left[ad\left(X\right)\right]y$ we have,

(42) $$z=x+y+\frac{1}{2}\left[ad\left(X\right)\right]y+\frac{1}{12}\left([ad(X)][ad(X)]y+[ad(Y)][ad(Y)]x\right)+\cdots ,$$

where the expansion in (41) is truncated to second-order with respect to X,Y, i.e., higher order terms involving [Y,[X,[Y,X]]] and [X,[Y,[X,Y]]], and above are neglected since once substituted in (39) and (40), they give rise to products of covariances or higher moments, which are assumed to be negligible. Using the definitions of mean and covariance in (39) and (40) for a convolved probability density u(g,t+δt)=u(g,t)∗u(g,δt), we obtain the following expressions for the mean and covariance of the convolved distribution correct to second order:

(43) μ(t+δt)=μ(t)∘μ(δt),

(44) $$\Sigma \left(t+\delta t\right)=\Sigma \left(\delta t\right)+\left[Ad\left(\mu {\left(\delta t\right)}^{-1}\right)\right]\Sigma \left(t\right){\left[Ad\left(\mu {\left(\delta t\right)}^{-1}\right)\right]}^{T}+F,$$

where *F* captures the second order propagation in the covariance. Its exact form is derived for SE(3) in [8] and more generally for unimodular groups in [11,33]. In this section, we concentrate on first order propagation since it admits a closed-form solution.

Successive compositions of μ(δt) using (43) provides the following closed form solution for the mean, which is obtained by integrating the exponent over time [12] (assuming that the initial condition is the identity element of the group):

(45) $$\mu \left(t\right)=\exp\left(\int_{0}^{t}m{\left(\tau \right)}^{\wedge }\;d\tau \right),$$

where m(τ) is defined in (35). Setting the initial condition to be the identity element does not lead to a loss of generality since the solution at any other initial condition can be obtained by convolving the fundamental solution (37) with a group Dirac delta function at the initial condition. Using Equation (41) we see that a successive composition becomes an integration in the exponent when the Lie bracket $[\int_{0}^{t}m{\left(\tau \right)}^{\wedge }d\tau ,m{\left(\delta t\right)}^{\wedge }\delta t]=0$ [7]; this holds in all examples considered in this paper since $m^{\wedge }$ is time-independent. Approximating Equation (44) to first order and recursively applying Equation (44) by discretising a domain from 0 to *t* into *n* segments with step-size δt and taking the limits n→∞ and δt→0, we obtain the following integral [12]:

(46) $$\Sigma \left(t\right)=\int_{0}^{t}\left[Ad\left(\mu {\left(\tau \right)}^{-1}\right)\right]D\left(\tau \right){\left[Ad\left(\mu {\left(\tau \right)}^{-1}\right)\right]}^{T}\;d\tau .$$

Then, an approximate solution to (35) can be constructed as,

(47) $$u\left(g,t\right)=u_{0}\left(g\right)*\frac{1}{{\left(2\pi \right)}^{N/2}{\left|\det\;\Sigma \left(t\right)\right|}^{1/2}}\exp\left(-\frac{1}{2}{\left[\log{\left(\mu {\left(t\right)}^{-1}\circ g\right)}^{\vee }\right]}^{T}\Sigma^{-1}\left(t\right)\left[\log{\left(\mu {\left(t\right)}^{-1}\circ g\right)}^{\vee }\right]\right),$$

where $u_{0}\left(g\right)$ is the initial condition. While initial conditions such as that for European put options [22] can also be considered, we focus on a Dirac delta initial condition centred at the group identity, $u_{0}\left(g\right)=\delta_{G}\left(g\right)$ since the solution for any other initial condition can be constructed by a convolution using (47). Moreover, the initial condition should be such that $u_{0}\left(g\right)$ is square-integrable in *G*.

### 4.1. Mean and Covariance Propagation for Diffusion Processes on $Gl^{+}\left(1\right)$

In this subsection, we apply the propagation technique to (23). An initial condition of $u_{0}\left(a\right)=\delta_{G}\left(a\right)=a\delta \left(a-1\right)$ is set (here δ(x) is the Euclidean Dirac delta distribution defined for $x\in R^{+}$). Using (45) and (46), we have $\mu \left(t^{\prime }\right)=\exp\left(-\left(r-\sigma^{2}/2\right)t^{\prime }\right)$ and $\Sigma \left(t^{\prime }\right)=\sigma^{2}t^{\prime }$. The expressions are in terms of reversed time $t^{\prime }=-t$. Substituting these expressions into (47) and noting that this is the fundamental solution $u_{f}\left(a,t^{\prime }\right)$, we have,

(48) $$u_{f}\left(a,t^{\prime }\right)=\frac{1}{\sigma \sqrt{2\pi t^{\prime }}}\exp\left(-\frac{1}{2\sigma^{2}t^{\prime }}{\left[\log a+\left(r-\frac{\sigma^{2}}{2}\right)t^{\prime }\right]}^{2}\right),$$

and the corresponding solution for the one-asset Black-Scholes Equation (9) would be $V_{f}\left(a,t^{\prime }\right)=u_{f}\left(a,t^{\prime }\right)e^{-rt^{\prime }}$. The solution obtained from covariance propagation exactly matches the standard analytical solution obtained after applying a logarithmic transformation x=loga to (9), as described in Appendix A.

### 4.2. Mean and Covariance Propagation for Diffusion Processes on $Gl^{+}\left(1\right)\times Gl^{+}\left(1\right)$

We now apply the propagation technique to (28) with an initial condition of $u_{0}\left(g\left(a,b\right)\right)=\delta_{G}\left(g\right)=ab\delta \left(a-1\right)\delta \left(b-1\right)$. Comparing (35) with (28), we have,

(49) $$m=-\left(\begin{array}{c} r-\sigma_{1}^{2}/2\\ r-\sigma_{2}^{2}/2 \end{array}\right)\;\;\mathrm{and}\;\;D=\left(\begin{array}{ccc} \sigma_{1}^{2} & \; & \rho \sigma_{1}\sigma_{2}\\ \rho \sigma_{1}\sigma_{2} & \; & \sigma_{2}^{2} \end{array}\right).$$

Then, the mean propagation equation would be,

(50) $$\mu \left(t^{\prime }\right)=\left(\begin{array}{ccc} \exp(-\left(r-\sigma_{1}^{2}/2\right)t^{\prime }) & \; & 0\\ 0 & \; & \exp(-\left(r-\sigma_{2}^{2}/2\right)t^{\prime }) \end{array}\right),$$

and $\Sigma \left(t^{\prime }\right)=Dt^{\prime }$. Substituting these expressions in (47), we obtain the fundamental solution $u_{f}\left(g\left(a,b\right),t^{\prime }\right)$ as,

(51) $$u_{f}\left(g\left(a,b\right),t^{\prime }\right)=\frac{1}{2\pi \sigma_{1}\sigma_{2}\sqrt{1-\rho^{2}}t^{\prime }}\exp\left(-\frac{1}{2\sigma_{1}^{2}\sigma_{2}^{2}\left(1-\rho^{2}\right)t^{\prime }}\left[\sigma_{1}^{2}l_{2}^{2}+\sigma_{2}^{2}l_{1}^{2}-2\rho \sigma_{1}\sigma_{2}l_{1}l_{2}\right]\right)$$

for $l_{1}\left(t^{\prime }\right)=\log a+\left(r-\sigma_{1}^{2}/2\right)t^{\prime }$ and $l_{2}\left(t^{\prime }\right)=\log b+\left(r-\sigma_{2}^{2}/2\right)t^{\prime }$. Propagation yields a solution that is equivalent to the standard solution obtained after applying the logarithmic transformations x=loga and y=logb to (12) as described in Appendix A. In summary, mean and covariance propagation applied to the one-asset and two-asset Black-Scholes models yields results that exactly match analytical solutions.

### 4.3. Mean and Covariance Propagation Requires Unimodularity of the Lie Group

Both $GL^{+}\left(1\right)$ and $GL^{+}\left(1\right)\times GL^{+}\left(1\right)$ are unimodular Lie groups. That is, the Haar measure dg for a group *G* is bi-invariant and is the only ‘natural’ measure (upto an arbitrary scaling by a constant) for which the following holds,

(52) $$\int_{G}f\left(g\right)dg=\int_{G}f\left(g\circ g_{0}\right)dg=\int_{G}f\left(g_{0}\circ g\right)dg=\int_{G}f\left(g^{-1}\right)dg,$$

for a fixed $g_{0}\in G$. However $Aff^{+}\left(1\right)$ is not a unimodular group, and one can define a right-invariant as well as a left-invariant Haar measure (but no bi-invariant measure exists). The theory of mean and covariance propagation in this section implicitly relies on the group being unimodular; this ensures that there is a unique bi-invariant Haar measure with respect to which a probability density function can be defined. If the group was not unimodular, a probability density defined with respect to one measure may not be a density with respect to the other. Therefore, to apply mean and covariance propagation to obtain the solution for (34), one approach would be to map the diffusion on the group to a diffusion on a related but unimodular group. In the next section, we show that that cotangent bundle of a group, when equipped with semidirect product group operation, is unimodular. This property will be exploited thereon by reinterpreting (34) as a diffusion on the cotangent bundle of the affine group.

## 5. Unimodularity of the Cotangent Bundle Group

Let *G* be an *N* dimensional matrix Lie group and the Lie algebra G is the tangent space of the group at identity with *N* basis vectors, represented by $E_{i}$ for i=1,⋯,N. The tangent bundle TG can be constructed and equipped with a group operation □ as,

(53) (TG,□)=G⋉G.

Similarly, the cotangent bundle, which is the dual space to the tangent bundle is $TG^{*}$, and can be equipped with the group operation ▪ as,

(54) $$\left(TG^{*},▪\right)=G⋉G^{*},$$

where there exists a bijection between G and $R^{N}$ and similarly between $G^{*}$ and $R^{N}$. Note that the two expressions indicate that the tangent and cotangent bundles have been endowed with a semidirect product. For an element g∈G and X∈G, the corresponding element in the tangent bundle would be (g,X) and the group operation in the tangent bundle will be,

(55) $$\left(g_{1},X_{1}\right)□\left(g_{2},X_{2}\right)=\left(g_{1}\circ g_{2},Ad\left(g_{1}\right)X_{2}+X_{1}\right)$$

where ∘ is the group operation in *G* and $Ad(g_{1})$ is the adjoint representation of $g_{1}\in G$ where Ad(g) is defined such that $Ad\left(g\right)X=gXg^{-1}$ for X∈G. If we were to represent Ad(g) as matrix, we have [Ad(g)] and the coadjoint representation would be ${\left[Ad\left(g\right)\right]}^{-T}$. This is because for X∈G and $Y\in G^{*}$, we can construct a bijection such that $X^{\vee }=x$ and $Y^{\vee }=y$ where $x,y\in R^{N}$. Note that the ∨ operator for elements from G and elements from $G^{*}$ are different but its usage will be clear from the object to which it is applied to. The inner product is defined to be $\left(X,Y\right)=x^{T}y=k$, k∈R. If we use the adjoint representation of the group, a typical element of the tangent space would be Ad(g)X and ${\left(Ad\left(g\right)X\right)}^{\vee }=\left[Ad\left(g\right)\right]x$. Then, the image of the cotangent space on $R^{N}$ would transform as ${\left[Ad\left(g\right)\right]}^{-T}y$ since ${\left(\left[Ad\left(g\right)\right]x\right)}^{T}\left({\left[Ad\left(g\right)\right]}^{-T}y\right)=k$. This motivates the use of ${\left[Ad\left(g\right)\right]}^{-T}$ as the dual to the adjoint representation in (55). Then, for $\left(g,Y\right)\in G⋉G^{*}$, we have,

(56) $$\left(g_{1},Y_{1}\right)▪\left(g_{2},Y_{2}\right)=\left(g_{1}\circ g_{2},Ad{\left(g_{1}\right)}^{-T}Y_{2}+Y_{1}\right),$$

where ${\left(Ad{\left(g\right)}^{-T}Y\right)}^{\vee }={\left[Ad\left(g\right)\right]}^{-T}y$ for $Y\in G^{*}$ and $Y^{\vee }=y\in R^{N}$. Tangent and cotangent bundles, endowed with these operations have been used before in [42].

A matrix representation can be constructed for both the tangent and cotangent bundles incorporating the semidirect product as,

(57) $$G⋉G≐\left\{\left.\left(\begin{array}{ccc} \left[Ad(g)\right] & \; & x\\ 0^{T} & \; & 1 \end{array}\right)\right|g\in G\;\mathrm{and}\;x\in G^{\vee }\right\},$$

and,

(58) $$G⋉G^{*}≐\left\{\left.\left(\begin{array}{ccc} {\left[Ad\left(g\right)\right]}^{-T} & \; & y\\ 0^{T} & \; & 1 \end{array}\right)\right|g\in G\;\mathrm{and}\;y\in {\left(G^{*}\right)}^{\vee }\right\}.$$

The dimension of the cotangent and tangent bundle group would be 2N provided that the adjoint representation Ad(G) is *N*-dimensional. This may not hold in certain cases, such as for GL(1), where Ad(G) is zero dimensional. More generally, these constructions will result in a 2N-dimensional semidirect product group if the group *G* has a trivial center. For the affine group $Aff^{+}\left(1\right)$, whose cotangent bundle group will be considered in the paper, this is true. However for G=GL(N), the center is one-dimensional (consisting of scalar multiples of the identity matrix $I_{N}$) and Ad(G) would be $N^{2}-1$ dimensional. We postpone the discussion of constructing the cotangent and tangent bundle groups for groups with non-trivial centers to a future paper.

### 5.1. Properties of the Adjoint Operator

The proofs of unimodularity will depend on the properties of the adjoint operator in the tangent and cotangent bundle group. We present a few properties that will be useful in constructing the proofs in the later subsections.

#### 5.1.1. $\left[Ad\left(g\right)\right]\left[ad\left(E_{i}\right)\right]\left[Ad\left(g^{-1}\right)\right]=\left[ad\left(Ad\left(g\right)E_{i}\right)\right]$

Since $Ad\left(g\right)E_{i}=gE_{i}g^{-1}$ and $ad\left(E_{i}\right)E_{j}=\left[E_{i},E_{j}\right]=E_{i}E_{j}-E_{j}E_{i}$, we can convert the matrix representation of the operator into its coordinate free form through the ∧ operator. For $x\in R^{N}$ where $x^{\wedge }=X\in G$, we have,

(59) $${\left(\left[Ad\left(g\right)\right]\left[ad\left(E_{i}\right)\right]\left[Ad\left(g^{-1}\right)\right]x\right)}^{\wedge }=Ad\left(g\right)ad\left(E_{i}\right)Ad\left(g^{-1}\right)X.$$

The right-hand side can be simplified as,

$$\begin{array}{cc} Ad\left(g\right)ad\left(E_{i}\right)Ad\left(g^{-1}\right)X & =Ad\left(g\right)ad\left(E_{i}\right)\left(g^{-1}Xg\right)\\ \; & =g\left[E_{i}g^{-1}Xg-g^{-1}XgE_{i}\right]g^{-1}\\ \; & =gE_{i}g^{-1}X-XgE_{i}g^{-1}\\ \; & =ad(gE_{i}g^{-1})X\\ \; & =ad(Ad\left(g\right)E_{i})X. \end{array}$$

Hence,

(60) $$\left[Ad\left(g\right)\right]\left[ad\left(E_{i}\right)\right]\left[Ad\left(g^{-1}\right)\right]x={\left(ad\left(Ad\left(g\right)E_{i}\right)X\right)}^{\vee }=\left[ad\left(Ad\left(g\right)E_{i}\right)\right]x.$$

This proves that $\left[Ad\left(g\right)\right]\left[ad\left(E_{i}\right)\right]\left[Ad\left(g^{-1}\right)\right]=\left[ad\left(Ad\left(g\right)E_{i}\right)\right]$ since it must hold for all $x\in G^{\vee }$.

#### 5.1.2. ${\left[ad\left(Ad\left(g\right)E_{i}\right)\right]}^{\vee }=\left[Ad\left(g\right)\right]e_{i}$

The relationship ${\left[ad\left(Ad\left(g\right)E_{i}\right)\right]}^{\vee }=\left[Ad\left(g\right)\right]e_{i}$ would follow if $E_{i}=e_{i}^{\wedge }$ and ${\left[ad\left(E_{i}\right)\right]}^{\vee }=E_{i}^{\vee }=e_{i}$. The ∨ operator defined on $\left[ad\left(E_{i}\right)\right]$ maps the Lie algebra of the tangent bundle to $R^{2N}$ whereas the ∨ operator acting on $E_{i}$ maps G to $R^{N}$. The overloading of the ∨ symbol should not lead to confusion since the object on which it acts will determine its interpretation.

### 5.2. Lie Algebra of TG and $TG^{*}$

The basis vectors spanning the Lie algebra of the tangent bundle can be obtained by differentiating an element representation (57) with respect to each of the *N* variables parameterizing it, at identity. The Lie algebra basis can be obtained for the parameters parameterizing the group *G* as well as for the parameters parameterizing G as,

(61) $${\tilde{E}}_{i}=\left\{\left(\begin{array}{ccc} \left[ad\left(E_{i}\right)\right] & \; & 0\\ 0^{T} & \; & 0 \end{array}\right)\;\;\mathrm{for}\;i=1,\cdots ,N\;\;\mathrm{and}\;\;\left(\begin{array}{ccc} O & \; & e_{i-N}\\ 0^{T} & \; & 0 \end{array}\right)\;\;\mathrm{for}\;i=N+1,\cdots ,2N\right\}.$$

Note that $e_{j-N}^{\wedge }=E_{j-N}$ where $E_{j-N}$ is a basis of G.

For ${\tilde{E}}_{i}$ defined to be a basis element of the Lie algebra of (TG,□), the ∨ operator is defined such that ${\tilde{E}}_{i}^{\vee }=e_{i}$ where this time i=1,⋯,2N and the ∨ operator maps from this Lie algebra to $R^{2N}$. A similar definition can also be created for the Lie algebra of the cotangent bundle, $\left(TG^{*},▪\right)$. In this case, the basis of the Lie algebra can be deduced as,

(62) $${\tilde{E}}_{i}=\left\{\left(\begin{array}{ccc} -{\left[ad\left(E_{i}\right)\right]}^{T} & \; & 0\\ 0^{T} & \; & 0 \end{array}\right)\;\;\mathrm{for}\;i=1,\cdots ,N\;\;\mathrm{and}\;\;\left(\begin{array}{ccc} O & \; & e_{i-N}^{*}\\ 0^{T} & \; & 0 \end{array}\right)\;\;\mathrm{for}\;i=N+1,\cdots ,2N\right\}.$$

Here, $E_{i}\in G$ but $e_{i}^{*}\in {\left(G^{*}\right)}^{\vee }$, the latter of which is related to the cotangent space at identity. In general for an element of the Lie algebra of G⋉G, the vee operator is defined such that,

(63) $${\left(\begin{array}{ccc} \left[ad(X)\right] & \; & y\\ 0^{T} & \; & 0 \end{array}\right)}^{\vee }=\left(\begin{array}{c} X^{\vee }\\ y \end{array}\right),$$

where X∈G and $y\in G^{\vee }$. A similar definition exists for the cotangent bundle,

(64) $${\left(\begin{array}{ccc} -{\left[ad\left(X\right)\right]}^{T} & \; & y\\ 0^{T} & \; & 0 \end{array}\right)}^{\vee }=\left(\begin{array}{c} X^{\vee }\\ y \end{array}\right),$$

where X∈G and $y\in {\left(G^{*}\right)}^{\vee }$. The specific ∨ used will be clear from the context in terms of which argument it is applied to. The Lie algebra for the (co-)tangent bundle group defined this way is only 2N-dimensional for groups with trivial centers.

### 5.3. Unimodularity of TG and $TG^{*}$

**Theorem** **1.** *The tangent bundle group (TG,□)=G⋉G of an N-dimensional Lie group with a trivial center is a 2N-dimensional unimodular Lie group if and only if the group G is unimodular.*

**Proof.**  This can be proven by considering the adjoint representation of an element within the tangent bundle. The adjoint, $Ad\left(h\right){\tilde{E}}_{i}$ where h∈G⋉G, is given through matrix notation as,

(65) $${\left(Ad\left(h\right){\tilde{E}}_{i}\right)}^{\vee }=\left(\begin{array}{ccc} \left[Ad(g)\right] & \; & x\\ 0^{T} & \; & 1 \end{array}\right){\tilde{E}}_{i}\left(\begin{array}{ccc} {\left[Ad\left(g\right)\right]}^{-1} & \; & -{\left[Ad\left(g\right)\right]}^{-1}x\\ 0^{T} & \; & 1 \end{array}\right).$$

Solving this by substituting the two different forms of ${\tilde{E}}_{i}$ obtained from (61) and using the relationship that $\left[Ad\left(g\right)\right]\left[ad\left(E_{i}\right)\right]{\left[Ad\left(g^{-1}\right)\right]}^{\vee }=\left[Ad\left(g\right)\right]e_{i}$ from Section 5.1.1 and Section 5.1.2 we obtain,

(66) $$\left[Ad\left(h\right)\right]=\left(\begin{array}{ccc} \left[Ad(g)\right] & \; & O\\ {}* & \; & \left[Ad(g)\right] \end{array}\right),$$

where * denotes a matrix of the same size as [Ad(g)]. The determinant of the adjoint det[Ad(h)] is ${\left(\det\;\left[Ad\left(g\right)\right]\right)}^{2}$. A group is unimodular if and only if the absolute value of the determinant of the adjoint is equal to 1. In this case, it can be seen that G⋉G would be unimodular if and only if |det[Ad(g)]|=1 and dim(Ad(G))=2N if and only if Z(G)=e where *e* is the identity element of *G*.

If *G* has a non-trivial center Z(G), then for a k∈Z(G), one can decompose $g=h\left(q_{1},\cdots ,q_{m}\right)\circ k\left(q_{m+1},\cdots ,q_{N}\right)$ such that *h* and *k* commute. This is true since one can partition *G* such that any g∈G can be decomposed into g=h∘k where $h\in F_{G/Z(G)}$, k∈Z(G), and $F_{G/Z(G)}\subset G$ is the ‘fundamental domain’ [33] associated with the quotient group G/Z(G). The dimension of Ad(G) will then be dim(G)−dim(Z(G))=m. Therefore, the current construction of using Ad(G) to represent the group *G* will no longer be faithful when dim(Z(G))≠0 implying that since the theorem and proof are restricted to the special construction Ad(G)⋉G, they only hold in their current form for the case where *G* has a trivial center and Ad(G)⋉G is 2N dimensional.  □

**Theorem** **2.** *The cotangent bundle group $\left(TG^{*},▪\right)=G⋉G^{*}$ of an N-dimensional Lie group with a trivial center is always a 2N-dimensional unimodular Lie group independent of the unimodularity of G.*

**Proof.**  We construct a proof in a very similar way as that in Theorem 1. Here, we obtain the adjoint representation of the cotangent bundle in the following form for $h\in G⋉G^{*}$,

(67) $$\left[Ad\left(h\right)\right]=\left(\begin{array}{ccc} \left[Ad(g)\right] & \; & O\\ {}* & \; & {\left[Ad\left(g\right)\right]}^{-T} \end{array}\right).$$

Since det[Ad(h)]=1, this ensures that the cotangent bundle group is unimodular, independent of the unimodularity of *G*. The restriction to trivial centers follows from the fact that $Ad{\left(G\right)}^{-T}$ is only *N*-dimensional for groups with trivial centers and therefore $Ad^{-T}\left(G\right)⋉G$ will only have the same number of dimensions as the cotangent bundle with such a restriction.  □

## 6. Option Price Evolution with Coupled Assets as a Diffusion Process on the Cotangent Bundle of the Affine Group

Now we apply the results from the previous section to construct the cotangent bundle of the affine group. The motivation is to re-express Equation (34) in terms of the Lie derivatives of the cotangent bundle of $Aff^{+}\left(1\right)$. An element *h* from the cotangent bundle of the affine group, $h\in Ad{\left(Aff^{+}\left(1\right)\right)}^{-T}⋉R^{2}$, can be expressed in the following form using (31) and (58),

(68) $$h=\left(\begin{array}{ccc} 1 & b/a & x\\ 0 & 1/a & y\\ 0 & 0 & 1 \end{array}\right),$$

where $\left(a,b,x,y\right)\in R^{+}\times R^{3}$. Using (62), the orthonormal basis of the Lie algebra of this group, expressed in matrices, is

(69) $${\tilde{E}}_{1}=\left(\begin{array}{ccc} 0 & 0 & 0\\ 0 & -1 & 0\\ 0 & 0 & 0 \end{array}\right)\;\;,\;{\tilde{E}}_{2}=\left(\begin{array}{ccc} 0 & 1 & 0\\ 0 & 0 & 0\\ 0 & 0 & 0 \end{array}\right)\;,\;\;{\tilde{E}}_{3}=\left(\begin{array}{ccc} 0 & 0 & 1\\ 0 & 0 & 0\\ 0 & 0 & 0 \end{array}\right)\;\mathrm{and}\;\;{\tilde{E}}_{4}=\left(\begin{array}{ccc} 0 & 0 & 0\\ 0 & 0 & 1\\ 0 & 0 & 0 \end{array}\right).$$

For $X=x_{1}{\tilde{E}}_{1}+x_{2}{\tilde{E}}_{2}+x_{3}{\tilde{E}}_{3}+x_{4}{\tilde{E}}_{4}$, we have $\left(x_{1},x_{2},x_{3},x_{4}\right)\in R^{4}$, and hence we can construct a bijection from the Lie algebra to $R^{4}$ such that ${\tilde{E}}_{i}^{\vee }=e_{i}$ for i=1,2,3,4. We argue that although the number of dimensions is doubled when we consider the cotangent bundle, this has the benefit of making the group unimodular. To see this, consider the left and right Jacobians of the cotangent bundle group:

(70) $$\left[{\tilde{J}}_{L}\right]=\left(\begin{array}{cccc} 1/a & 0 & 0 & 0\\ -b/a & 1 & 0 & 0\\ by/a & -y & 1 & 0\\ y/a & 0 & 0 & 1 \end{array}\right)\;\;\mathrm{and}\;\;\left[{\tilde{J}}_{R}\right]=\left(\begin{array}{cccc} 1/a & 0 & 0 & 0\\ 0 & 1/a & 0 & 0\\ 0 & 0 & 1 & -b\\ 0 & 0 & 0 & a \end{array}\right)$$

Then, we observe that $\det\;{\tilde{J}}_{R}=\det\;{\tilde{J}}_{L}=1/a$, implying unimodularity. The adjoint of the affine cotangent bundle [Ad(h)] is,

(71) $$\left[Ad\left(h\right)\right]=\left(\begin{array}{ccccccc} 1 & \; & 0 & \; & 0 & \; & 0\\ -b & \; & a & \; & 0 & \; & 0\\ by & \; & -ay & \; & 1 & \; & b/a\\ y & \; & 0 & \; & 0 & \; & 1/a \end{array}\right),$$

where det[Ad(h)]=1, as expected. For completeness, [ad(X)] for $X=\sum_{i=1}^{4}x_{i}{\tilde{E}}_{i}$ is,

(72) $$\left[ad\left(X\right)\right]=\left(\begin{array}{ccccccc} 0 & \; & 0 & \; & 0 & \; & 0\\ -x_{2} & \; & x_{1} & \; & 0 & \; & 0\\ 0 & \; & -x_{4} & \; & 0 & \; & x_{2}\\ x_{4} & \; & 0 & \; & 0 & \; & -x_{1} \end{array}\right).$$

The left and right Lie derivatives are,

(73) $$\left(\begin{array}{c} {\tilde{E}}_{1}^{L}f\\ {\tilde{E}}_{2}^{L}f\\ {\tilde{E}}_{3}^{L}f\\ {\tilde{E}}_{4}^{L}f \end{array}\right)=\left(\begin{array}{c} a\;\partial \tilde{f}/\partial a+b\;\partial \tilde{f}/\partial b-y\;\partial \tilde{f}/\partial y\\ \partial \tilde{f}/\partial b+y\;\partial \tilde{f}/\partial x\\ \partial \tilde{f}/\partial x\\ \partial \tilde{f}/\partial y \end{array}\right)\;\;\mathrm{and}\;\;\left(\begin{array}{c} {\tilde{E}}_{1}^{R}f\\ {\tilde{E}}_{2}^{R}f\\ {\tilde{E}}_{3}^{R}f\\ {\tilde{E}}_{4}^{R}f \end{array}\right)=\left(\begin{array}{c} a\;\partial \tilde{f}/\partial a\\ a\;\partial \tilde{f}/\partial b\\ \partial \tilde{f}/\partial x\\ \left(b/a\right)\;\partial \tilde{f}/\partial x+\left(1/a\right)\;\partial \tilde{f}/\partial y \end{array}\right),$$

where $f\left(h\left(a,b,x,y\right)\right)=\tilde{f}\left(a,b,x,y\right)$. We now observe from (33) and (73) that ${\tilde{E}}_{1}^{R}=E_{1}^{R}$ and ${\tilde{E}}_{2}^{R}=E_{2}^{R}$, which allows us to rewrite (34) in terms of the cotangent bundle group Lie derivatives as,

(74) $$\frac{\partial u}{\partial t^{\prime }}=\left(r-\frac{\sigma_{1}^{2}}{2}\right){\tilde{E}}_{1}^{R}u+\left[\left(\frac{\mu_{2}}{\mu_{1}}-\frac{\sigma_{2}}{\sigma_{1}}\right)\mu_{1}+\frac{\sigma_{2}}{\sigma_{1}}r-\frac{\sigma_{1}\sigma_{2}}{2}\right]{\tilde{E}}_{2}^{R}u+\frac{1}{2}\left(\sigma_{1}^{2}{\left({\tilde{E}}_{1}^{R}\right)}^{2}+\sigma_{1}\sigma_{2}\left({\tilde{E}}_{2}^{R}{\tilde{E}}_{1}^{R}+{\tilde{E}}_{1}^{R}{\tilde{E}}_{2}^{R}\right)+\sigma_{2}^{2}{\left({\tilde{E}}_{2}^{R}\right)}^{2}\right)u,$$

which is a degenerate diffusion over the affine cotangent bundle group. We denote the ‘master’ function as, u=u(h(a,b,x,y),t), and $\tilde{u}=\tilde{u}\left(a,b,t\right)$ as the marginalised version of this ‘master’ function marginalised over the variables *x* and *y*.

### 6.1. Mean and Covariance Propagation for Diffusion Processes in the Cotangent Bundle of $Aff^{+}\left(1\right)$

Since $Ad{\left(Aff^{+}\left(1\right)\right)}^{-T}⋉R^{2}$ is unimodular, the theory of mean and covariance propagation can be applied directly from Section 4 to solve (74). The initial condition is $u_{0}\left(h\left(a,b,x,y\right)\right)=\delta_{G}\left(h\right)=a\delta \left(a-1\right)\delta \left(b-1\right)\delta \left(x\right)\delta \left(y\right)$ where μ(0) would be the 2×2 identity matrix and the mean propagation equation would be,

(75) $$\mu \left(t^{\prime }\right)=\left(\begin{array}{ccc} 1 & -k_{2}\left(e^{k_{1}t^{\prime }}-1\right)/k_{1} & 0\\ 0 & \exp(k_{1}t^{\prime }) & 0\\ 0 & 0 & 1 \end{array}\right),$$

for $k_{1}=r-\sigma_{1}^{2}/2$ and $k_{2}=\left(\frac{\mu_{2}}{\mu_{1}}-\frac{\sigma_{2}}{\sigma_{1}}\right)\mu_{1}+\frac{\sigma_{2}}{\sigma_{1}}r-\frac{\sigma_{1}\sigma_{2}}{2}$.

Comparing (74) with (35), we have,

(76) $$D=\left(\begin{array}{ccccccc} \sigma_{1}^{2} & \; & \sigma_{1}\sigma_{2} & \; & 0 & \; & 0\\ \sigma_{1}\sigma_{2} & \; & \sigma_{2}^{2} & \; & 0 & \; & 0\\ 0 & \; & 0 & \; & 0 & \; & 0\\ 0 & \; & 0 & \; & 0 & \; & 0 \end{array}\right),$$

which is a singular matrix, implying that this is a degenerate diffusion over the cotangent bundle group. The covariance propagation equation is then,

(77) $$\Sigma \left(t^{\prime }\right)=\left(\begin{array}{ccc} \tilde{\Sigma }\left(t^{\prime }\right) & \; & O_{2}\\ O_{2} & \; & O_{2} \end{array}\right),$$

such that,

(78) $$\tilde{\Sigma }\left(t^{\prime }\right)=\left(\begin{array}{cc} \sigma_{1}^{2}t^{\prime } & \left[\sigma_{1}\sigma_{2}-\kappa \sigma_{1}^{2}\right]I_{1}\left(t^{\prime }\right)+\kappa \sigma_{1}^{2}t^{\prime }\\ \sigma_{1}\sigma_{2}-\kappa \sigma_{1}^{2}]I_{1}\left(t^{\prime }\right)+\kappa \sigma_{1}^{2}t^{\prime } & \left[\sigma_{1}^{2}\kappa^{2}-2\sigma_{1}\sigma_{2}\kappa +\sigma_{2}^{2}\right]I_{2}\left(t^{\prime }\right)+2\left[\sigma_{1}\sigma_{2}\kappa -\sigma_{1}^{2}\kappa^{2}\right]I_{1}\left(t^{\prime }\right)+\sigma_{1}^{2}\kappa^{2}t^{\prime } \end{array}\right),$$

where $\kappa =k_{2}/k_{1}$, $I_{1}=\left(e^{k_{1}t^{\prime }}-1\right)/k_{1}$ and $I_{2}=\left(e^{2k_{1}t^{\prime }}-1\right)/\left(2k_{1}\right)$. Noticeably $\Sigma (t^{\prime })$ is singular, but we treat this issue by writing,

(79) $$\Sigma \left(t^{\prime }\right)=\lim_{\epsilon \to 0}\Sigma_{\epsilon }\left(t^{\prime }\right)=\lim_{\epsilon \to 0}\left(\begin{array}{ccc} \tilde{\Sigma }\left(t^{\prime }\right) & \; & O_{2}\\ O_{2} & \; & \epsilon I_{2} \end{array}\right),$$

where $I_{2}$ is the 2 × 2 identity matrix. Then, $u_{f}\left(h\left(a,b,x,y\right),t^{\prime }\right)$ can be written as:

(80) $$u_{f}\left(h,t^{\prime }\right)=\lim_{\epsilon \to 0}\left(\frac{1}{{\left(2\pi \right)}^{2}{\left|\det\;\Sigma_{\epsilon }\left(t^{\prime }\right)\right|}^{1/2}}\exp\left(-\frac{1}{2}{\left[\log{\left(\mu {\left(t^{\prime }\right)}^{-1}\circ h\right)}^{\vee }\right]}^{T}\Sigma_{\epsilon }^{-1}\left(t^{\prime }\right)\left[\log{\left(\mu {\left(t^{\prime }\right)}^{-1}\circ h\right)}^{\vee }\right]\right)\right).$$

For the affine cotangent bundle, it is possible to obtain a closed-form expression for $\log{\left(\mu {\left(t^{\prime }\right)}^{-1}\circ h\right)}^{\vee }$. Any $\mu \left(t^{\prime }\right)\in Ad{\left(Aff^{+}\left(1\right)\right)}^{-T}⋉R^{2}$ can be expressed in the following form,

(81) $$\mu \left(t^{\prime }\right)=\left(\begin{array}{ccccc} 1 & \; & b^{\prime }/a^{\prime } & \; & 0\\ 0 & \; & 1/a^{\prime } & \; & 0\\ 0 & \; & 0 & \; & 1 \end{array}\right),$$

using the expression of *h* in (68) and where $a^{\prime }$ and $b^{\prime }$ are functions of $t^{\prime }$. Then, one has,

(82) $$\log{\left(\mu^{-1}\circ h\right)}^{\vee }=\left(\begin{array}{c} -\log(a^{\prime }/a)\\ -\log\left(a^{\prime }/a\right)\left(b-b^{\prime }\right)/\left(a-a^{\prime }\right)\\ F_{1}\\ F_{2} \end{array}\right),$$

where,

(83) $$F_{1}=x+\left(\frac{\left(a^{\prime }b-b^{\prime }a\right)}{a-a^{\prime }}+\frac{aa^{\prime }\log\left(a/a^{\prime }\right)\left(b-b^{\prime }\right)}{{\left(a-a^{\prime }\right)}^{2}}\right)y$$

(84) $$F_{2}=\frac{aa^{\prime }\log\left(a/a^{\prime }\right)}{a-a^{\prime }}y.$$

The derivations of these expressions are provided in Appendix D. For convenience of notation, we express the first two components of $\log{\left(\mu {\left(t^{\prime }\right)}^{-1}\circ h\right)}^{\vee }$ as $\bar{\log}{\left(\mu {\left(t^{\prime }\right)}^{-1}\circ h\right)}^{\vee }$ and the third and fourth components with F so that we have,

(85) $$\log{\left(\mu {\left(t^{\prime }\right)}^{-1}\circ h\right)}^{\vee }=\left(\begin{array}{c} \bar{\log}{\left(\mu {\left(t^{\prime }\right)}^{-1}\circ h\right)}^{\vee }\\ F \end{array}\right).$$

Substituting this form into (80) and taking the limit ϵ→0, we obtain,

(86) $$u_{f}\left(h,t^{\prime }\right)=\frac{1}{\left(2\pi \right)|\det\;\tilde{\Sigma }\left(t^{\prime }\right){|}^{1/2}}\exp\left(-\frac{1}{2}{\left[\bar{\log}{\left(\mu {\left(t^{\prime }\right)}^{-1}\circ h\right)}^{\vee }\right]}^{T}{\tilde{\Sigma }}^{-1}\left(t^{\prime }\right)\left[\bar{\log}{\left(\mu {\left(t^{\prime }\right)}^{-1}\circ h\right)}^{\vee }\right]\right)\delta \left(F_{1}\right)\delta \left(F_{2}\right),$$

where δ(⋯) is the Dirac delta function. Additionally, we re-express $\delta \left(F_{1}\right)\delta \left(F_{2}\right)$ in terms of δ(x)δ(y) as,

(87) $$\delta \left(F_{1}\right)\delta \left(F_{2}\right)=\left|\frac{a-a^{\prime }}{aa^{\prime }\log\left(a/a^{\prime }\right)}\right|\delta \left(x\right)\delta \left(y\right),$$

which can be derived by calculating the Jacobian determinant of the system of Equations in (83) and (84). We note that the variables *x* and *y* are extraneous to the original problem and we aim to marginalise the distribution $u_{f}\left(h\left(a,b,x,y\right),t^{\prime }\right)$ from (80) to ${\tilde{u}}_{f}\left(a,b,t^{\prime }\right)$ as,

(88) $${\tilde{u}}_{f}\left(a,b,t^{\prime }\right)=\int_{R}\int_{R}u_{f}\left(h\left(a,b,x,y\right),t^{\prime }\right)\;dx\;dy.$$

Using (87) and (86), we have,

(89) $${\tilde{u}}_{f}\left(a,b,t^{\prime }\right)=\frac{1}{\left(2\pi \right)|\det\;\tilde{\Sigma }\left(t^{\prime }\right){|}^{1/2}}\exp\left(-\frac{1}{2}{\left[\bar{\log}{\left(\mu {\left(t^{\prime }\right)}^{-1}\circ h\right)}^{\vee }\right]}^{T}{\tilde{\Sigma }}^{-1}\left(t^{\prime }\right)\left[\bar{\log}{\left(\mu {\left(t^{\prime }\right)}^{-1}\circ h\right)}^{\vee }\right]\right)\left|\frac{a-a^{\prime }}{aa^{\prime }\log\left(a/a^{\prime }\right)}\right|,$$

which is the fundamental solution to (74).

### 6.2. Normalisation of Probability Distribution Functions on the Affine Cotangent Bundle Group

For the affine cotangent bundle group, we can define a general Gaussian as,

(90) $$u\left(h,t^{\prime }\right)=c\left(\Sigma \right)\exp\left(-\frac{1}{2}{\left[\log{\left(\mu^{-1}\left(t^{\prime }\right)\circ h\right)}^{\vee }\right]}^{T}\Sigma^{-1}\left[\log{\left(\mu^{-1}\left(t^{\prime }\right)\circ h\right)}^{\vee }\right]\right),$$

for $h\in Aff^{+}\left(1\right)⋉R^{2}$, where a general normalisation factor c(Σ), which is a function of the covariance Σ, is used. In the previous section, we used a factor of the form, $c\left(\Sigma_{\epsilon }\right)={\left|\det\;\Sigma_{\epsilon }^{-1}\right|}^{1/2}/{\left(2\pi \right)}^{N/2}$ for N=4 in the affine cotangent bundle group (prior to marginalisation; see Equation (80)). However, this is only correct for small covariances and assuming that the determinant of the group Jacobian (70) is sufficiently close to 1. The goal of this section is to derive a higher order correction to this normalisation factor for the affine cotangent bundle group. To do so, we first convert to exponential coordinates so that for an arbitrary element $g\in Aff^{+}\left(1\right)⋉R^{2}$,

(91) $$\log{\left(g\right)}^{\vee }=q=\left(\begin{array}{c} \alpha \\ \beta \\ \gamma \\ \phi \end{array}\right),$$

where $q={\left[\alpha ,\beta ,\gamma ,\phi \right]}^{T}$ is the exponential coordinate parameterization of the group. Then, the right Jacobian of the group defined in (16) for this coordinate system would be

(92) $${\left[J_{R}\right]}_{\exp}=\left(\begin{array}{ccccccc} (1/a)\partial a/\partial \alpha & \; & 0 & \; & 0 & \; & 0\\ (1/a)\partial b/\partial \alpha & \; & (1/a)\partial b/\partial \beta & \; & 0 & \; & 0\\ \partial x/\partial \alpha -b\partial y/\partial \alpha & \; & \partial x/\partial \beta & \; & \partial x/\partial \gamma & \; & \partial x/\partial \phi -b\partial y/\partial \phi \\ a\partial y/\partial \alpha & \; & 0 & \; & 0 & \; & a\partial y/\partial \phi \end{array}\right),$$

where the use of (a,b,x,y) makes contact with the earlier parameterization in (68); expressing α,β,γ and ϕ as functions of (a,b,x,y) we have,

(93) $$\begin{array}{cc} \alpha (a) & =\log a,\\ \beta (a,b) & =\frac{\log a}{a-1}b,\\ \gamma (a,b,x,y) & =x+by\left(\frac{1}{a-1}-\frac{\log a}{{\left(a-1\right)}^{2}}\right),\\ \phi (a,y) & =\frac{a\log a}{a-1}y. \end{array}$$

Then, we have,

(94) $$\det\;J_{\exp}=\frac{1}{e^{\alpha }}\frac{\partial a}{\partial \alpha }\frac{\partial b}{\partial \beta }\frac{\partial x}{\partial \gamma }\frac{\partial y}{\partial \phi }=\frac{{\left(e^{\alpha }-1\right)}^{2}}{\alpha^{2}e^{\alpha }},$$

where since the cotangent bundle group is unimodular, we have dropped the *R* subscript noting that $\det\;J_{R}=\det\;J_{L}$ independent of the parameterization. Expanding $\det\;J_{\exp}$ to small α we have,

(95) $$\det\;J_{\exp}=1+\frac{1}{12}\alpha^{2}+O\left(\alpha^{4}\right).$$

We can also write this relation as,

(96) $$\det\;J_{\exp}\left(q\right)\approx 1-\frac{1}{2}q^{T}Wq,$$

for

(97) $$W=\left(\begin{array}{ccccccc} -1/6 & \; & 0 & \; & 0 & \; & 0\\ 0 & \; & 0 & \; & 0 & \; & 0\\ 0 & \; & 0 & \; & 0 & \; & 0\\ 0 & \; & 0 & \; & 0 & \; & 0 \end{array}\right).$$

Since to $O(\alpha^{4})$,

(98) $$\exp\left(-\frac{1}{2}q^{T}Wq\right)\approx 1-\frac{1}{2}q^{T}Wq,$$

we have

(99) $$\det\;J_{\exp}\approx \exp\left(-\frac{1}{2}q^{T}Wq\right).$$

Now if we consider the integral of (90) over the cotangent bundle group,

(100) $$\int_{G}u\left(h,t^{\prime }\right)\;dh=1,$$

and make the substitution $g=\mu^{-1}\circ h$, we can rewrite (100) as,

(101) $$\int_{G}c\left(\Sigma \right)\exp\left(-\frac{1}{2}q^{T}\Sigma^{-1}q\right)\;dg=1,$$

by making use of the unimodularity of the cotangent bundle and noting that $\log{\left(g\left(q\right)\right)}^{\vee }=q$ for a parameterization in terms of the exponential coordinates q. Thus,

(102) $$\begin{array}{cc} \int_{G}c\left(\Sigma \right)\exp\left(-\frac{1}{2}q^{T}\Sigma^{-1}q\right)\;dg & =\int_{q}c\left(\Sigma \right)\exp\left(-\frac{1}{2}q^{T}\Sigma^{-1}q\right)\;\det\;J_{\exp}\left(q\right)\;dq\\ \; & \approx c\left(\Sigma \right)\int_{q}\exp\left(-\frac{1}{2}q^{T}\left(\Sigma^{-1}+W\right)q\right)\;dq, \end{array}$$

where we use the relationship for the determinant of the Jacobian in (99). Equating (102) to the number 1 and noting that the integral evaluates to ${\left(2\pi \right)}^{\frac{N}{2}}/{\left|\det\;\left(\Sigma^{-1}+W\right)\right|}^{1/2}$, we have,

(103) $$c\left(\Sigma \right)\approx \frac{|\det\;\left(\Sigma^{-1}+W\right){|}^{\frac{1}{2}}}{{\left(2\pi \right)}^{2}}.$$

In the context of the degenerate diffusions on the affine cotangent bundle group that arise in the coupled asset model, we have,

(104) $$c\left(\tilde{\Sigma }\right)\approx \frac{|\det\;\left({\tilde{\Sigma }}^{-1}+\tilde{W}\right){|}^{\frac{1}{2}}}{\left(2\pi \right)},$$

for

(105) $$\tilde{W}=\left(\begin{array}{ccc} -1/6 & \; & 0\\ 0 & \; & 0 \end{array}\right).$$

We also note that the sign of the non-zero element of $\tilde{W}$ is negative. Whereas, all terms of ${\tilde{\Sigma }}^{-1}$ are positive. This suggests that at a sufficiently large covariance, it is possible that $c(\tilde{\Sigma })=0$ as a consequence of the current approximation. While this was not observed for the small covariances used in this paper, this suggests an opportunity to use a different method of approximating the integral,

$$\int_{q}c\left(\Sigma \right)\exp\left(-\frac{1}{2}q^{T}\Sigma^{-1}q\right)\;\det\;J_{\exp}\left(q\right)\;dq,$$

by using the following general relation for $q\in R^{N}$ [29,43]:

(106) $$\int_{q}q^{T}Wq\exp\left(-\frac{1}{2}q^{T}\Sigma^{-1}q\right)\;dq={\left(2\pi \right)}^{N/2}\frac{\mathrm{tr}(W\Sigma )}{|\det\;\Sigma^{-1}{|}^{\frac{1}{2}}},$$

where tr(⋯) is the trace operator. Nevertheless, in this paper we do not pursue this approximation and instead use the result in (104) assuming that the eigenvalues of ${\tilde{\Sigma }}^{-1}$ are sufficiently large (which is the case for the range of parameters used in the numerical simulations in the subsequent sections).

## 7. Numerical Results for Option Price Evolution with Coupled Assets

We solve the PDE in (74) by four methods: (1) Finite difference method (implicit and explicit), (2) first order propagation, (3) second order propagation and (4) Euler–Maruyama integration of the underlying stochastic differential equations. The theory of second order covariance propagation has not yet been introduced for $Aff^{+}\left(1\right)⋉R^{2}$, and will also be described in this section. All simulations were performed using MATLAB R2019a on a 2.7 GHz Dual-Core Intel Core i5 processor. The CPU time to run 10 time steps of second order propagation, explicit finite difference method and implicit finite difference method was 18.94 s, 0.99 s and 11.55 s (assuming that the finite difference matrices are constructed before-hand), respectively; however, if the matrix logarithms are evaluated analytically rather than numerically—which is possible for the affine cotangent bundle group (82)—the CPU time for first order propagation reduces to 0.30 s for 10 time steps.

### 7.1. Finite Difference Method

The 2D finite difference scheme was implemented on a rectangular *a*–*b* grid with second-order accuracy. Explicit and implicit schemes were constructed, using the forward and backward Euler scheme, respectively. A time-step of $10^{-6}$ units was used for the explicit scheme and a time-step of $10^{-3}$ units for the implicit scheme. A smaller time-step was used for the explicit method to ensure stability. A grid spacing of approximately $4.92\times 10^{-3}$ units along *a* and $5.24\times 10^{-3}$ units along *b* was used. The simulation domain was chosen to best capture the distribution while ensuring that the boundaries B were sufficiently far from the mode of the distribution. This was to ensure that the Dirichlet boundary condition of $\tilde{u}{\left(a,b\right)|}_{B}=0$ could be set. A Dirac delta initial condition was approximated using a circular Gaussian with a standard deviation equal to twice the grid spacing along *a*.

### 7.2. Euler–Maruyama Integration of Underlying Stochastic Differential Equations

It is possible to convert a Fokker–Planck equation over an *N*-dimensional Lie group to a stochastic differential equation in the Lie algebra. A Fokker–Planck equation for a probability density of the form u(g,t) in the group *G* with a drift of m(t) and diffusivity D(t) is,

(107) $$\frac{\partial u}{\partial t}=-\sum_{i=1}^{N}m_{i}\left(t\right){\tilde{E}}_{i}^{R}u+\sum_{i,j=1}^{N}D_{ij}\left(t\right){\tilde{E}}_{i}^{R}{\tilde{E}}_{j}^{R}u,$$

where ${\tilde{E}}_{i}^{R}$ is a right directional Lie derivative in *G*. From [17,29], we recognise that the stochastic differential equation described by this Fokker-Planck equation on the Lie algebra G would be of the form,

(108) $$d\tilde{x}=m\left(t\right)dt+B\left(t\right)dW,$$

where dW is a vector of increments of *N* uncorrelated Wiener processes, $W_{1},\cdots ,W_{N}$, corresponding to random draws from a Gaussian with zero mean and variance dt, and $BB^{T}=D$. Equation (108) can be interpreted either as a Stratonovich or Itô equation since the diffusion term *B* is independent of $\tilde{x}$. Since $\tilde{x}\in G^{\vee }$, this process occurs in the Lie algebra of *G*. This process can then be injected on to the group [17] as,

(109) $$g\left(t+dt\right)=g\left(t\right)\circ \exp\left(d{\tilde{x}}^{\wedge }\right),$$

thereby defining a stochastic process on *G*. If we were to parameterize the group elements with a vector $q\in R^{N}$ such that $d\tilde{x}=\left[{\tilde{J}}_{R}\left(q\right)\right]dq$ where $\left[{\tilde{J}}_{R}\left(q\right)\right]$ is the right Jacobian matrix, we obtain a Stratonovich stochastic differential equation in the parameter space $R^{N}$ as,

(110) $$dq={\left[{\tilde{J}}_{R}\right]}^{-1}\left(m\left(t\right)dt+B\left(t\right)\;Ⓢ\;dW\right),$$

where Ⓢ emphasises that this is a Stratonovich equation. For the cotangent bundle group of the affine group, $dq={\left[da,db,dx,dy\right]}^{T}$ and the form of $\left[{\tilde{J}}_{R}\right]$ is provided in (70). Matching (107) with (74), we obtain,

(111) $$m=-\left(\begin{array}{c} \left(r-\sigma_{1}^{2}/2\right)\\ \left(\mu_{2}/\mu_{1}-\sigma_{2}/\sigma_{1}\right)\mu_{1}+\sigma_{2}r/\sigma_{1}-\sigma_{1}\sigma_{2}/2\\ 0\\ 0 \end{array}\right),$$

and,

(112) $$B=\frac{1}{\sqrt{\sigma_{1}^{2}+\sigma_{2}^{2}}}\left(\begin{array}{ccccccc} \sigma_{1}^{2} & \; & \sigma_{1}\sigma_{2} & \; & 0 & \; & 0\\ \sigma_{1}\sigma_{2} & \; & \sigma_{2}^{2} & \; & 0 & \; & 0\\ 0 & \; & 0 & \; & 0 & \; & 0\\ 0 & \; & 0 & \; & 0 & \; & 0 \end{array}\right).$$

Using the form of $\left[{\tilde{J}}_{R}\right]$ from (70) and the expressions for m and *B* from (111) and (112), we have the following Stratonovich stochastic differential equations for a,b,x,y:

(113) $$\left(\begin{array}{c} da\\ db\\ dx\\ dy \end{array}\right)=\left(\begin{array}{c} a(m_{1}dt+B_{11}\;Ⓢ\;dW_{1}+B_{12}\;Ⓢ\;dW_{2})\\ a(m_{2}dt+B_{21}\;Ⓢ\;dW_{1}+B_{22}\;Ⓢ\;dW_{2})\\ 0\\ 0 \end{array}\right),$$

where dx=0 and dy=0 highlight the degenerate nature of the stochastic differential equation. However, to implement an Euler-Maruyama integration in parameter space, we require the stochastic differential equations to be expressed as Itô equations; the distinction between the Itô and Stratonovich form of a stochastic differential equation is especially important here since the diffusivity is now a function of *a*. The Itô version of the equations is thus,

(114) $$\left(\begin{array}{c} da\\ db\\ dx\\ dy \end{array}\right)=\left(\begin{array}{c} a\left(\left(m_{1}+\frac{1}{2}\left(B_{11}^{2}+B_{12}^{2}\right)\right)dt+B_{11}dW_{1}+B_{12}dW_{2}\right)\\ a\left(\left(m_{2}+\frac{1}{2}\left(B_{21}B_{11}+B_{22}B_{12}\right)\right)dt+B_{21}dW_{1}+B_{22}dW_{2}\right)\\ 0\\ 0 \end{array}\right),$$

where the terms $\frac{1}{2}\left(B_{11}^{2}+B_{12}^{2}\right)$ and $\frac{1}{2}\left(B_{21}B_{11}+B_{22}B_{12}\right)$ correct for the drift. Equivalently, instead of solving (114), it is also possible to obtain an evolution in parameter space by projecting the stochastic evolution of g(a(t),b(t),x(t),y(t)) in the cotangent bundle (109) on to the space of parameters (a,b,x,y). This is because for any $g\left(t\right)\in Aff^{+}\left(1\right)⋉R^{2}$ we have,

(115) $$g\left(t\right)=g\left(a\left(t\right),b\left(t\right),x\left(t\right),y\left(t\right)\right)=\left(\begin{array}{ccccc} 1 & \; & b(t)/a(t) & \; & x(t)\\ 0 & \; & 1/a(t) & \; & y(t)\\ 0 & \; & 0 & \; & 1 \end{array}\right),$$

which determines a unique point in a stochastic trajectory in parameter space (a(t),b(t),x(t),y(t)). Due to the degenerate nature of the diffusion process, there is no evolution in the parameters (x,y). This method of obtaining the stochastic process in parameter space, although equivalent in principle to that obtained by integrating (114), is henceforth referred to as an Itô-Gangolli method since it makes use of the McKean-Gangolli injection [17] and numerically solves the stochastic differential equations in the Lie algebra of the group (108) by an Euler-Maruyama integration.

The group-theoretic covariance and mean of the probability density function u(g,t) were deduced from the ensemble generated by (109) on the group using the methods in [8,33]. That is, the discrete version of (39) can be obtained by setting the sampled probability density to be $u_{s}\left(g,t\right)=\frac{1}{N}\sum_{i=1}^{N_{s}}\delta_{G}\left(gg_{i}^{-1}\right)$ for a total of $N_{s}$ samples. Here, $\delta_{G}\left(g\right)$ is the group Dirac delta function. A similar substitution in (40) gives the sample covariance. Thus, the sampled mean $\mu_{s}\left(t\right)$ and covariance $\Sigma_{s}\left(t\right)$ are,

(116) $$\sum_{i=1}^{N_{s}}{\left[\log\left(\mu_{s}{\left(t\right)}^{-1}\circ g_{i}\left(t\right)\right)\right]}^{\vee }=0,$$

(117) $$\Sigma_{s}\left(t\right)=\frac{1}{N_{s}}\sum_{i=1}^{N_{s}}\left[\log{\left(\mu_{s}{\left(t\right)}^{-1}\circ g_{i}\left(t\right)\right)}^{\vee }\right]{\left[\log{\left(\mu_{s}{\left(t\right)}^{-1}\circ g_{i}\left(t\right)\right)}^{\vee }\right]}^{T}.$$

In the context of the Euler–Maruyama integration, $N_{s}$ is the total number of sample paths and the averaging is performed at each time slice *t*. The value of $g_{i}\left(t\right)$ is obtained from the evolution process in *G* described in (109). One would expect that for large $N_{s}$, $\mu_{s}\left(t\right)$ and $\Sigma_{s}\left(t\right)$ will approximate the corresponding mean and covariance of the solution of (74) to the extent that the exact solution remains a Gaussian on the cotangent bundle group. Hence, the results from the Euler-Maruyama integration of a large number of sample paths can serve as a baseline truth to compare results against. Note that (116) needs to be solved iteratively by beginning with the following guess solution,

(118) $$\mu_{s}^{0}\left(t\right)=\sum_{i=1}^{N_{s}}{\left[\log\left(g_{i}\left(t\right)\right)\right]}^{\vee },$$

and the mean at the ${\left(j+1\right)}^{\mathrm{th}}$ iteration can be computed using,

(119) $$\mu_{s}^{j+1}\left(t\right)=\mu_{s}^{j}\left(t\right)\circ \exp\left(\frac{1}{N_{s}}\sum_{i=1}^{N_{s}}\left[\log\left(\mu_{s}^{j}{\left(t\right)}^{-1}\circ g_{i}\left(t\right)\right)\right]\right),$$

where we observe that $\frac{1}{N_{s}}\sum_{i=1}^{N_{s}}\left[\log\left(\mu_{s}^{j}{\left(t\right)}^{-1}\circ g_{i}\left(t\right)\right)\right]$ quantifies an error that goes to zero once the mean converges. This converged value is substituted as $\mu_{s}\left(t\right)$ in (117) to obtain the sampled covariance. In the simulations, $N_{s}$ = 30,000 and a time-step of $10^{-3}$ units was used. After generating the ensemble of sample paths, a continuous probability distribution was created in (a,b) space by kernel density estimation using a Gaussian kernel. This kernel density estimated probability distribution was used as a baseline to compare against finite difference and propagation solutions (shown later in the contour plots of Figure 3 and Figure 6).

### 7.3. Second Order Propagation for $Aff^{+}\left(1\right)⋉R^{2}$

The mean propagates by (43) where $\mu \left(\delta t\right)=\exp\left(m^{\wedge }\delta t\right)$ for $m=-{\left[k_{1},k_{2}\right]}^{T}$ using the definitions in (75). Using (72) and (42) and substituting it in the definition of covariance in (40), we have the following expression for second-order covariance propagation [11,33]:

(120) Σ(t+δt)=A+B+F(A,B),

where,

(121) $$\begin{array}{cc} \; & A=\left[Ad\left(\mu {\left(\delta t\right)}^{-1}\right)\right]\Sigma \left(t\right){\left[Ad\left(\mu {\left(\delta t\right)}^{-1}\right)\right]}^{T},\\ \; & B=D\delta t,\\ \; & F\left(A,B\right)=\frac{1}{4}C+\frac{1}{12}\left(P+P^{T}+Q+Q^{T}\right), \end{array}$$

for

$$C=\sum_{i,j=1}^{4}[ad\left({\tilde{E}}_{i}\right]B[ad\left({\tilde{E}}_{j}\right]A_{ij}\;\;,\;\;P=\left(\sum_{i,j=1}^{4}[ad\left({\tilde{E}}_{i}\right][ad\left({\tilde{E}}_{j}\right]A_{ij}\right)B\;\;\mathrm{and}\;\;Q=\left(\sum_{i,j=1}^{4}[ad\left({\tilde{E}}_{i}\right][ad\left({\tilde{E}}_{j}\right]B_{ij}\right)A.$$

**Figure 1:**
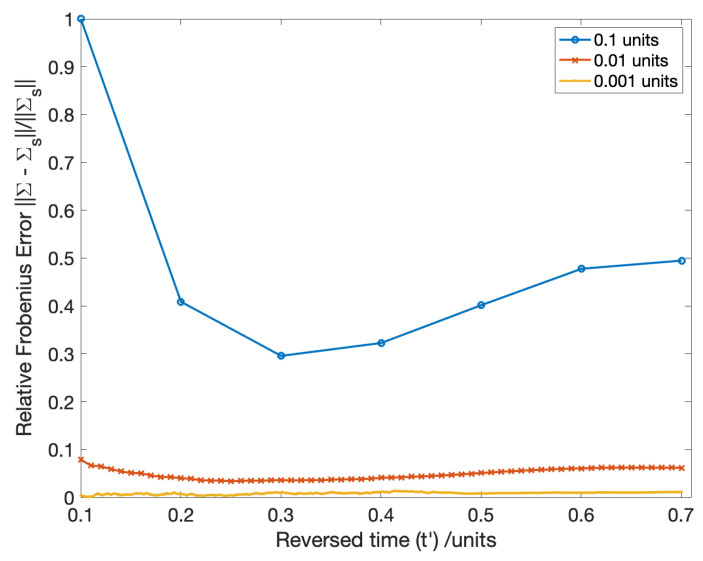
Convergence of second order propagated Σ with reducing time-step, relative to the sample standard deviation $\Sigma_{s}\left(t^{\prime }\right)$. The horizontal axis shows the reversed time $t^{\prime }$ from 0.1 to 0.7 units.

Much like the case for first order propagation, only the first 2×2 subspace of $\Sigma (t^{\prime })$ is non-zero, corresponding to $\tilde{\Sigma }\left(t^{\prime }\right)$. A time-step of $10^{-3}$ units was used in second order propagation. Figure 1 depicts the convergence of the error in $\Sigma (t^{\prime })$ with respect to time-step. The baseline truth in this case was $\Sigma_{s}\left(t^{\prime }\right)$ sampled from the ensemble of paths generated in the Euler-Maruyama integration (117). The parameters used in this study were: $\sigma_{1}=0.1$, $\sigma_{2}=0.05$, r=3, $\mu_{1}=1$ and $\mu_{2}=2$. To compare the covariances, the metric in (122) was used.

### 7.4. Results

For the numerical simulations, we consider the PDE in (74) with two sets of parameters, $S_{l}:\left\{\sigma_{1}=0.1,\;\sigma_{2}=0.05,\;r=3,\;\mu_{1}=1,\;\mu_{2}=2\right\}$ and $S_{h}:\left\{\sigma_{1}=1,\;\sigma_{2}=0.5,\;r=3,\;\mu_{1}=1,\;\mu_{2}=2\right\}$; the set of parameters $S_{l}$ describes a scenario where diffusion of asset price is low and $S_{h}$ describes a scenario where the diffusion is relatively large. Since the values of $\mu_{1}$, $\mu_{2}$ and *r* are same in both cases, the values of $\sigma_{1}$ and $\sigma_{2}$ will be used to distinguish these two sets. The constraint $\sigma_{2}/\sigma_{1}=\left(\mu_{2}-r\right)/\left(\mu_{1}-r\right)$ was used to fix the ratio $\sigma_{2}/\sigma_{1}$ in both cases. Additionally, the initial condition is a Dirac delta distribution at the identity element of the cotangent bundle group of $Aff^{+}\left(1\right)$.

**Figure 2:**
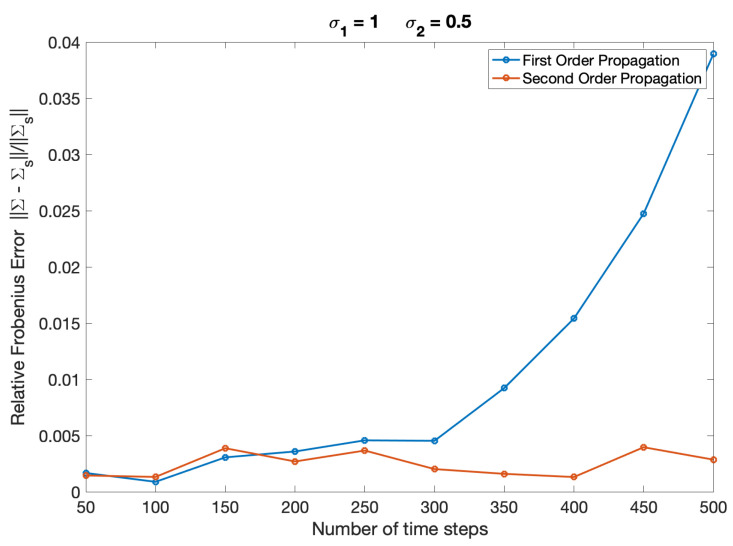
Relative error comparing $\Sigma (t^{\prime })$ at a given time-step with the sampled covariance $\Sigma_{s}\left(t^{\prime }\right)$ for parameter values $\sigma_{1}=1$ and $\sigma_{2}=0.5$, representing a scenario with large diffusion.

Firstly, we show the differences between first and second order group-theoretic covariance propagation for the large diffusion scenario ($S_{h}$) in Figure 2. The error in covariance is plotted as a relative deviation using the following error metric,

(122) $$\mathrm{Error}\;(t^{\prime })=\frac{||\Sigma \left(t^{\prime }\right)-\Sigma_{s}\left(t^{\prime }\right)\left|\right|}{\left|\right|\Sigma_{s}\left(t^{\prime }\right)\left|\right|},$$

where ||⋯|| denotes the Frobenius matrix norm, $\Sigma_{s}\left(t^{\prime }\right)$ is the sampled covariance from (117) and $\Sigma (t^{\prime })$ is either the first or second order propagated covariance.

The relative error in mean was evaluated in a similar fashion:

(123) $$\mathrm{Error}\;(t^{\prime })=\frac{||\mu \left(t^{\prime }\right)-\mu_{s}\left(t^{\prime }\right)\left|\right|}{\left|\right|\mu_{s}\left(t^{\prime }\right)\left|\right|},$$

where $\mu_{s}\left(t^{\prime }\right)$ is the sampled mean from (116) and $\mu (t^{\prime })$ is either the first or second order propagated mean. For the set of parameters describing ‘large diffusion’ (parameter set $S_{h}$) the relative error in the mean was approximately 0.1 to 0.2% and first order and second order mean propagation were indistinguishable. Furthermore, in the case of ‘small diffusion’ (parameter set $S_{l}$), the error in covariance was approximately 0.4 to 1.2% and with minimal difference between first order and second order propagated results; finally, the relative error in mean for this set of parameters was in the order of 0.02% and again with no difference between first and second order propagation. Only approximate values are given since this range of error is within the variability of the sampled mean and covariance itself, which are used as the baseline.

We now proceed to compare the results from first order and second order propagation with those from finite-difference methods, relative to the probability density function obtained from the Itô-Gangolli method used to indirectly solve the stochastic differential equations in (114). The probability distribution corresponding to the ensemble of points $\left(a,b\right)\in R^{+}\times R$ in parameter space was considered as the ground truth for the following numerical studies. One can then estimate the mean and covariance of this ground truth at time $t^{\prime }$ by,

(124) $$\begin{array}{cc} \mu_{s}\left(t^{\prime }\right) & =\frac{1}{N_{s}}\sum_{i=1}^{N_{s}}q_{i} \end{array}$$

(125) $$\begin{array}{cc} \Sigma_{s}\left(t^{\prime }\right) & =\frac{1}{N_{s}}\sum_{i=1}^{N_{s}}\left(q_{i}-\mu \left(t^{\prime }\right)\right){\left(q_{i}-\mu \left(t^{\prime }\right)\right)}^{T} \end{array}$$

for $N_{s}$ sample paths and $q_{i}={\left[a_{i}\left(t\right),b_{i}\left(t\right)\right]}^{T}$. Note that these are the expressions for the mean and covariance defined in Euclidean space (see Appendix C). It is important to emphasise that here we are *not* comparing on the basis of the group-theoretic mean and covariance but rather on the basis of a mean and covariance defined in $R^{+}\times R^{3}$. Since the solution from propagation (followed by marginalisation) or finite difference methods would yield a probability density over the affine cotangent bundle group, it is important to convert these results to an equivalent probability density function on parameter space. That is, if f(g,t) is a probability distribution on the affine cotangent bundle,

(126) $$\int_{G}f\left(g,t\right)\;dg=\int_{q}\tilde{f}\left(q,t\right)\;\det\;J\left(q\right)\;dq=1,$$

and $\tilde{\tilde{f}}\left(q,t\right)=\tilde{f}\left(q,t\right)\;\det\;J\left(q\right)$ would be a probability distribution in parameter space $R^{+}\times R^{3}$. In the special case of degenerate diffusion for the coupled asset model, $\tilde{\tilde{f}}\left(q,t\right)$ is a probability distribution in the Euclidean half-space $R^{+}\times R$. The mean and covariance of such a distribution can then be evaluated as,

(127) $$\begin{array}{cc} \mu (t) & =\int_{q}q\tilde{\tilde{f}}\left(q,t\right)\;dq \end{array}$$

(128) $$\begin{array}{cc} \Sigma (t) & =\int_{q}\left(q-\mu \right){\left(q-\mu \right)}^{T}\tilde{\tilde{f}}\left(q,t\right)\;dq. \end{array}$$

For $q={\left[a,b\right]}^{T}$; we also have detJ(q)=1/a from (70). The mean and covariance defined this way is evaluated for the finite difference and propagation solution (based on (89) but using the higher-order normalisation factor from (104)) and compared against the sampled mean and covariance obtained from the ground truth in (124,125).

#### 7.4.1. Small Diffusion: $\sigma_{1}=0.1$, $\sigma_{2}=0.05$

**Figure 3:**
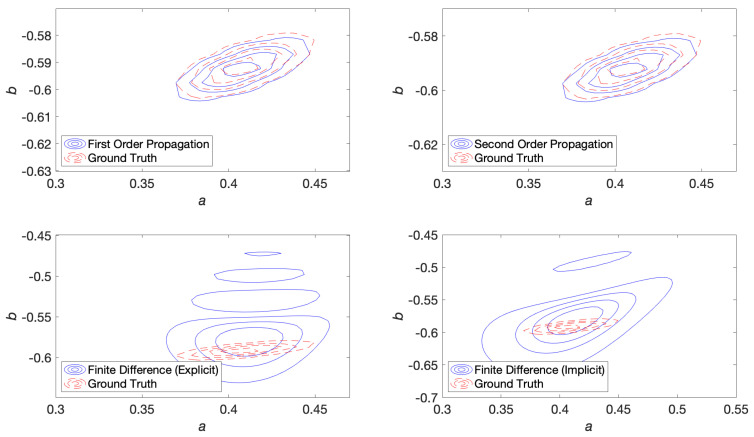
Contour plots at 300 time steps into the simulation ($t^{\prime }=0.30$) for the small diffusion scenario, showing the close match between the first order and second order propagation against the ground truth (kernel estimated probability density) but a worse match for the finite difference solutions.

Contour plots were generated for first order propagation, second order propagation, explicit and implicit finite difference methods. The baseline probability density was smoothed by a kernel density estimation procedure using a Gaussian kernel, which was used as the ground truth for the contour plots to qualitatively assess the shape of the distribution. These are shown at 300 time steps into the simulation in Figure 3.

**Figure 4:**
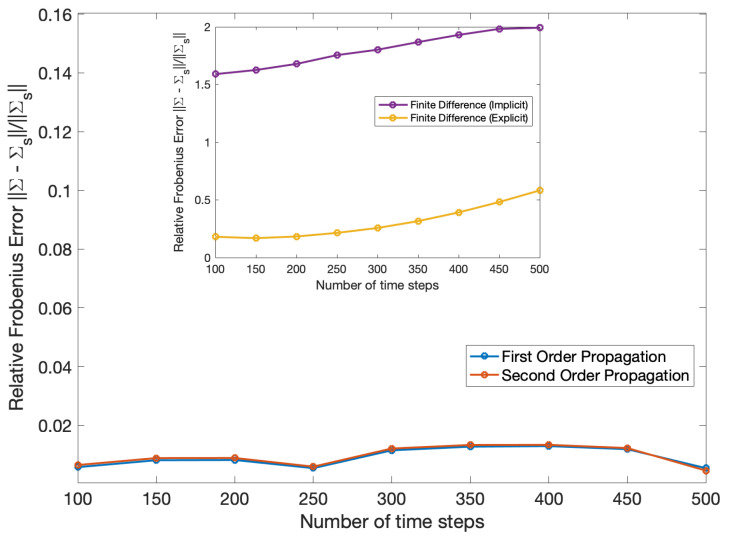
Relative error in covariance: Comparison between first order propagation, second order propagation and explicit and implicit finite difference (inset) for the small diffusion scenario. The propagation results nearly coincide and therefore cannot be distinguished in the plot.

The poor performance of the finite difference solution relative to the propagation solution is also evident in Figure 4. In this figure, the relative error is measured with respect to the sampled covariance (125) and calculated using the same formula in (122), but where $\Sigma (t^{\prime })$ is the covariance in terms of parameters (a,b) from (128).

**Figure 5:**
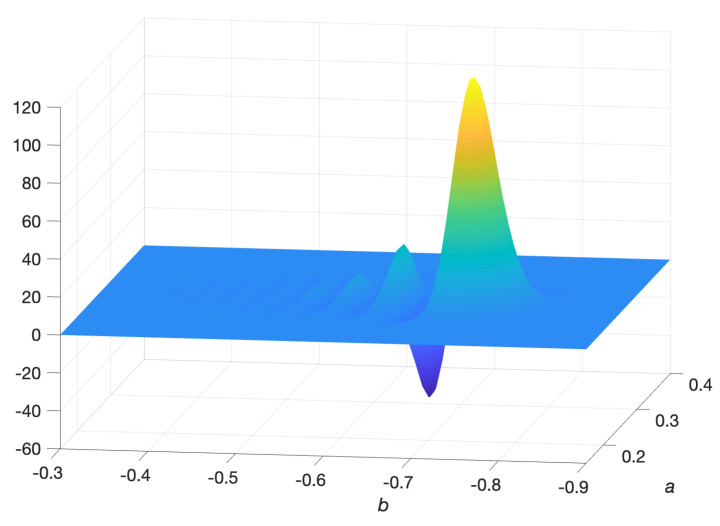
Finite difference solution (explicit) at 450 time steps ($t^{\prime }=0.45$) showing spurious oscillations due to the small covariances in the small diffusion scenario.

The relatively poor performance of the finite difference solution is because the covariance is very small (but not zero) such that one observes spurious oscillations in the finite difference solution (see Figure 5). Negative values of ${\tilde{u}}_{f}\left(a,b,t\right)$ are artefacts of the discretisation. In simulating the evolution of distributions with low covariance, a finite difference solution requires a very fine mesh near the mode of the distribution to avoid such artefacts whereas a propagated solution requires no such discretisation in (a,b) space and automatically avoids these issues. Nevertheless, the relative error in mean, measured through a Euclidean norm, was approximately 0.2 to 0.4% for both propagation and finite difference simulations.

#### 7.4.2. Large Diffusion: $\sigma_{1}=1$, $\sigma_{2}=0.5$

**Figure 6:**
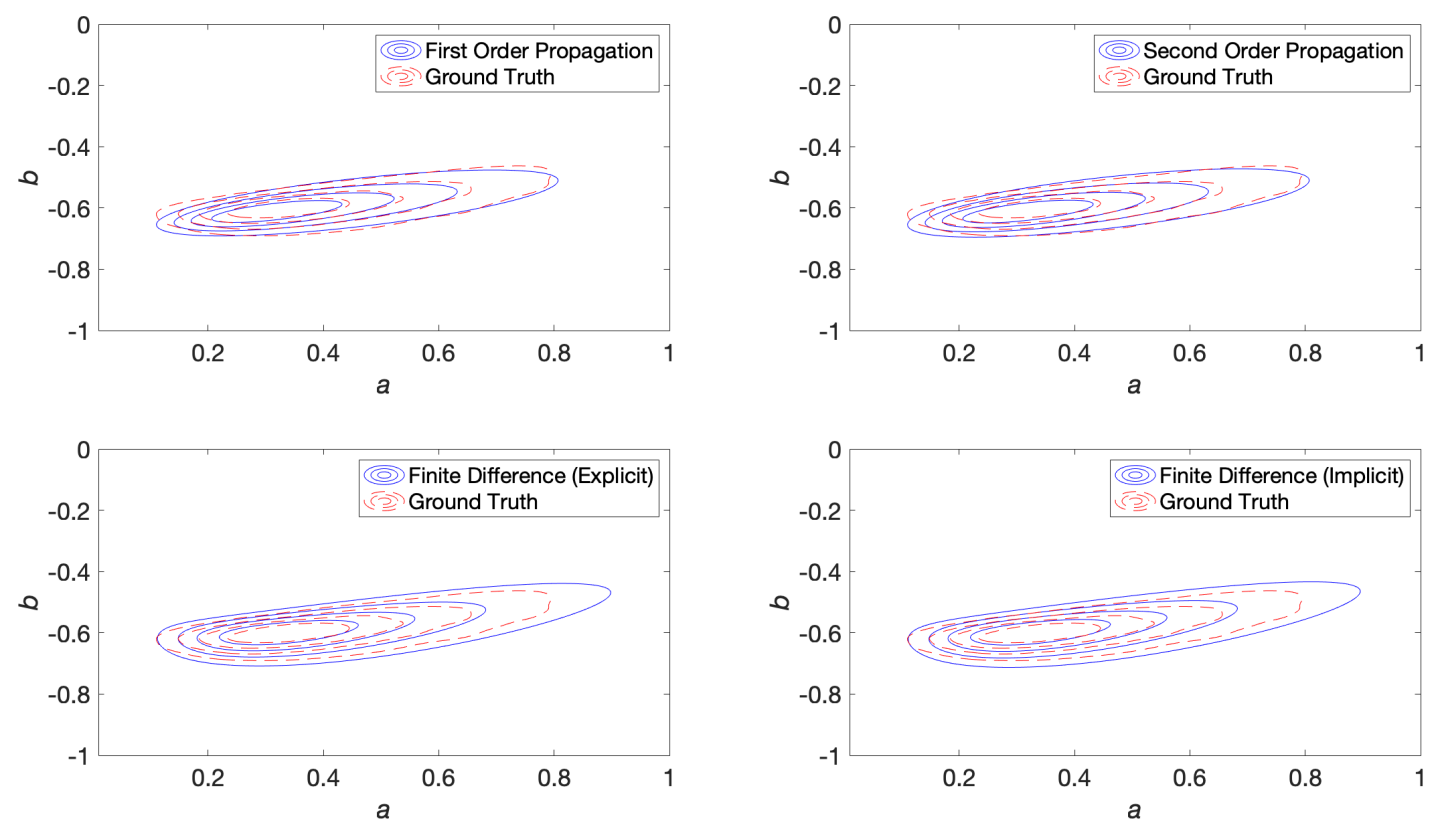
Contour plots at 300 time steps into the simulation ($t^{\prime }=0.30$) for the large diffusion scenario, with the kernel density estimated probability distribution used as the ground truth.

Contour plots were generated for first order propagation, second order propagation, explicit and implicit finite difference methods. These are shown at 300 time steps into the simulation in Figure 6.

**Figure 7:**
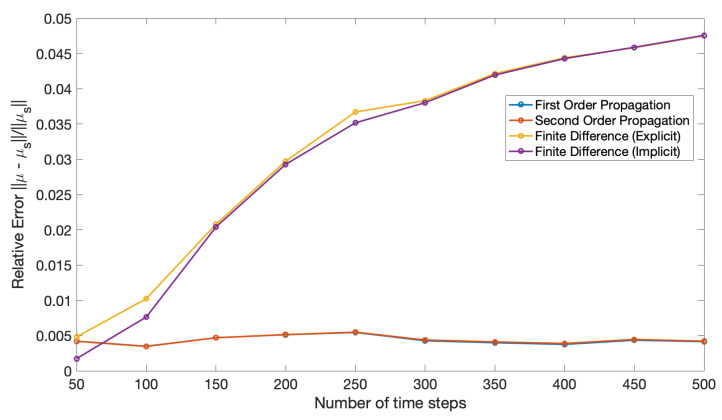
Relative error in mean: Comparison between first order, second order propagation and finite difference methods (explicit and implicit) for the large diffusion scenario. The propagation results nearly coincide and therefore cannot be distinguished in the plot.

**Figure 8:**
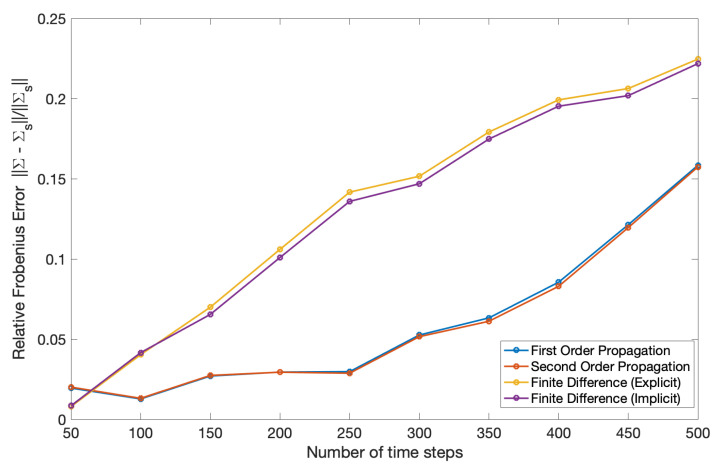
Relative error in covariance: Comparison between first order, second order propagation and finite difference methods (explicit and implicit) for the large diffusion scenario.

Figure 7 and Figure 8 show the mean and covariance evaluated using (127) and (128) compared relative to (124) and (125). The error in covariance was measured by a relative Frobenius norm and the relative error in mean was measured using a Euclidean norm defined as,

(129) $$\frac{\left||\epsilon |\right|}{\left|\right|\mu_{s}\left|\right|}=\sqrt{\frac{\epsilon^{T}\epsilon }{\mu_{s}^{T}\mu_{s}}},$$

for $\epsilon =\mu -\mu_{s}$ and $\mu_{s}$ is the sampled mean (124).

Mean and covariance propagation when applied to the coupled asset model tend to yield results with lower error when the covariances are small, in line with the original assumptions made to derive the technique. Additionally, covariance propagation is more suitable in dealing with Dirac delta initial conditions than a finite difference method. Another advantage of the propagation technique as opposed to a finite difference solution is that there is no grid involved: one effectively has a solution that is not discretized in the (a,b) domain and only requires a temporal discretization (for the second order propagation) or no discretization at all (for the first order propagation where the solution can be written in closed-form). Finally, a synergy of the two methods would be useful in spanning a broader range of covariances than either method can handle on its own.

## 8. Backward Compatibility of Propagation on the Cotangent Bundle with the One-Asset Black-Scholes Equation

In this final section, we consider reframing the one-asset Black-Scholes equation as a diffusion process on the affine cotangent bundle group. This is possible because the Lie derivative of $GL^{+}\left(1\right)$ (22) can also be represented by ${\tilde{E}}_{1}^{r}$ from (73). Hence, we can write (24) in terms of the affine cotangent bundle group Lie derivatives as,

(130) $$\frac{\partial u}{\partial t^{\prime }}=\left(r-\frac{\sigma^{2}}{2}\right){\tilde{E}}_{1}^{R}u+\frac{\sigma^{2}}{2}{\tilde{E}}_{1}^{R}u.$$

By first order propagation, we know that the mean is (assuming an identity initial condition),

(131) $$\mu \left(t^{\prime }\right)=\exp\left(-\left(r-\frac{\sigma^{2}}{2}\right){\tilde{E}}_{1}t^{\prime }\right)=\left(\begin{array}{ccccc} 1 & \; & 0 & \; & 0\\ 0 & \; & \exp(\left(r-\frac{\sigma^{2}}{2}\right)t^{\prime }) & \; & 0\\ 0 & \; & 0 & \; & 1 \end{array}\right).$$

The diffusion matrix is now,

(132) $$D=\left(\begin{array}{ccccccc} \sigma^{2} & \; & 0 & \; & 0 & \; & 0\\ 0 & \; & 0 & \; & 0 & \; & 0\\ 0 & \; & 0 & \; & 0 & \; & 0\\ 0 & \; & 0 & \; & 0 & \; & 0 \end{array}\right),$$

and,

(133) $$\Sigma \left(t^{\prime }\right)=\int_{0}^{t^{\prime }}[Ad\left(\mu^{-1}\left(\tau \right)\right]D[Ad{\left(\mu^{-1}\left(\tau \right)\right]}^{T}\;d\tau =\left(\begin{array}{ccccccc} \sigma^{2}t^{\prime } & \; & 0 & \; & 0 & \; & 0\\ 0 & \; & 0 & \; & 0 & \; & 0\\ 0 & \; & 0 & \; & 0 & \; & 0\\ 0 & \; & 0 & \; & 0 & \; & 0 \end{array}\right),$$

since $\left[Ad\left(\mu {\left(t^{\prime }\right)}^{-1}\right)\right]$ and *D* are both diagonal. Similar results can also be obtained numerically using second order propagation for both mean and covariance. Following the discussion in Section 6.1, we construct a Gaussian on the cotangent bundle of the form,

(134) $$u_{f}\left(h\left(a,b,x,y\right),t^{\prime }\right)=\frac{1}{\sigma \sqrt{2\pi t^{\prime }}}\exp\left(\frac{-1}{2\sigma^{2}t^{\prime }}{\left[\bar{\log}{\left(\mu {\left(t^{\prime }\right)}^{-1}\circ h\right)}^{\vee }\right]}^{T}\left[\bar{\log}{\left(\mu {\left(t^{\prime }\right)}^{-1}\circ h\right)}^{\vee }\right]\right)\delta \left(F_{b}\right)\delta \left(F_{1}\right)\delta \left(F_{2}\right),$$

where using the form of $\mu (t^{\prime })$ in (81) we have,

(135) $$\mu \left(t^{\prime }\right)=\left(\begin{array}{ccccc} 1 & \; & 0 & \; & 0\\ 0 & \; & 1/a^{\prime } & \; & 0\\ 0 & \; & 0 & \; & 1 \end{array}\right),$$

so that,

(136) $$F_{b}=-\frac{\log(a^{\prime }/a)}{a-a^{\prime }}b$$

(137) $$F_{1}=x+\left(\frac{a^{\prime }}{a-a^{\prime }}+\frac{aa^{\prime }\log\left(a/a^{\prime }\right)}{{\left(a-a^{\prime }\right)}^{2}}\right)by$$

(138) $$F_{2}=\frac{aa^{\prime }\log\left(a/a^{\prime }\right)}{a-a^{\prime }}y,$$

and $\bar{\log}{\left(\mu {\left(t^{\prime }\right)}^{-1}\circ h\right)}^{\vee }=\log{\left(\mu {\left(t^{\prime }\right)}^{-1}\circ h\right)}^{\vee }\cdot e_{1}$. Then we can show that,

(139) $$\delta \left(F_{b}\right)\delta \left(F_{1}\right)\delta \left(F_{2}\right)=\frac{\delta (b)\delta (x)\delta (y)}{\det\;J^{\prime }}=\frac{1}{aa^{\prime }}{\left(\frac{a-a^{\prime }}{\log(a^{\prime }/a)}\right)}^{2}\delta \left(b\right)\delta \left(x\right)\delta \left(y\right)$$

by constructing a Jacobian matrix $J^{\prime }$ from the system of equations in (136)–(138) and using its determinant $\det\;J^{\prime }$ to normalise the Dirac delta function. Marginalising over the variables b,x,y, we obtain the solution for the one-asset Black-Scholes equation given by propagation on the affine cotangent bundle as,

(140) $$u_{f}\left(h\left(a,b,x,y\right),t^{\prime }\right)=\frac{1}{\sigma \sqrt{2\pi t^{\prime }}}\exp\left(\frac{-1}{2\sigma^{2}t^{\prime }}{\left[\left(r-\frac{\sigma^{2}}{2}\right)t^{\prime }+\log a\right]}^{2}\right)\frac{1}{aa^{\prime }}{\left(\frac{a-a^{\prime }}{\log(a^{\prime }/a)}\right)}^{2}.$$

**Figure 9:**
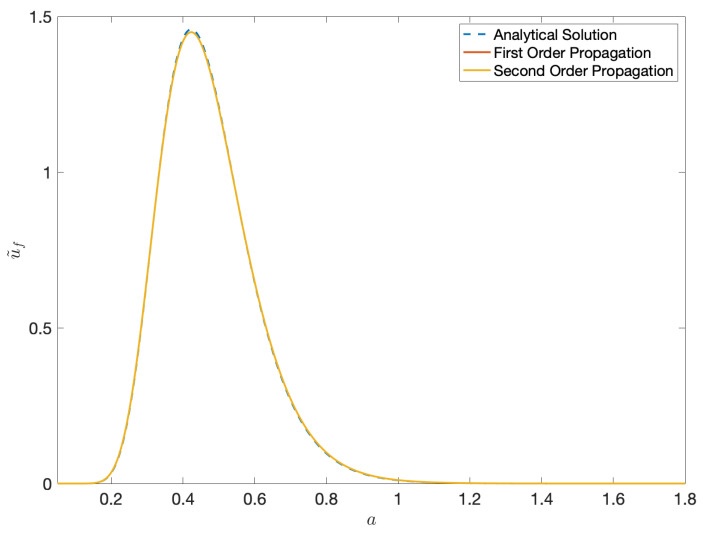
Plot of the analytical and propagated solution to the converted 1D Black-Scholes equation in (130), ${\tilde{u}}_{f}\left(a,t^{\prime }\right)$, for the small diffusion case (σ=0.5, r=3 and $t^{\prime }=0.3$). Both first order and second order propagation results coincide.

**Figure 10:**
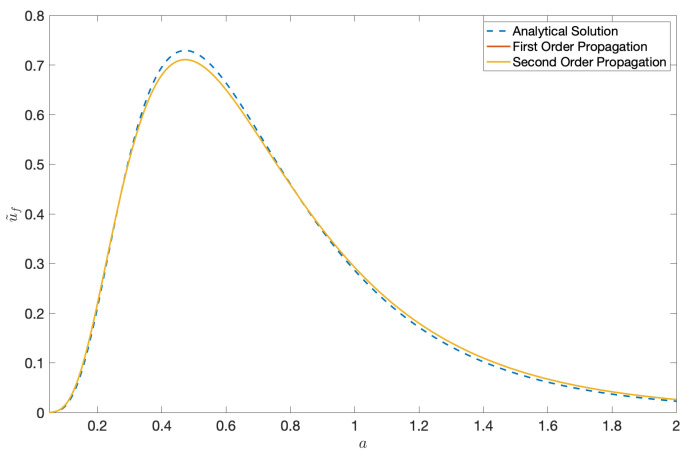
Plot of the analytical and propagated solution to the converted 1D Black-Scholes equation in (130), ${\tilde{u}}_{f}\left(a,t^{\prime }\right)$, for the large diffusion case (σ=1, r=3 and $t^{\prime }=0.3$). Both first order and second order propagation results coincide.

It is important to note that the solution in (140) differs from (48) by the Jacobian factor $1/\det\;J^{\prime }$ and therefore is not an exact solution. However we see that for small values of $\epsilon =\left(a-a^{\prime }\right)/a^{\prime }$, $1/\det\;J^{\prime }\approx 1$ to $O(\epsilon^{2})$. In this case, it is also possible to compare the propagated result with the analytical solution for the Black-Scholes equation. We make this comparison for $S_{l}:\left\{r=3,\;\sigma =0.5\right\}$ representing a small diffusion scenario and $S_{h}:\left\{r=3,\;\sigma =1\right\}$ representing a large diffusion scenario, at $t^{\prime }=0.30$ and show the plots in Figure 9 and Figure 10. Moreover, the higher-order normalisation factor in (104) is used to normalise the propagated result in (140) instead of $1/\sigma \sqrt{2\pi t^{\prime }}$. We see a closer match with the analytical solution for the small diffusion scenario.

## 9. Conclusions

Reframing PDEs in $R^{N}$ as diffusion processes on Lie groups offers an alternative method to solve PDEs by using mean and covariance propagation techniques developed previously in the context of Fokker–Planck equations on Lie groups [6,7,8,9,10,12]. In the case of asset dynamics from mathematical finance, the method yields the exact solution for the one-asset and two-asset problems by matching the Lie derivatives of the one-asset and two-asset Black-Scholes equations with the Lie derivatives of $GL^{+}\left(1\right)$ and $GL^{+}\left(1\right)\times GL^{+}\left(1\right)$, respectively; this trivially reduces to the logarithmic coordinate transformation that converts these equations to heat equations.

While using the apparatus of mean/covariance propagation on Lie groups is undue for the one-asset and two-asset Black-Scholes equations, the matching is especially useful for the model of option price evolution under coupled asset dynamics introduced in the paper where the logarithmic coordinate transformation characteristic of the one-asset and two-asset Black-Scholes PDE can no longer be applied. Instead, we solve the equation by matching the derivatives with the Lie derivatives of $Aff^{+}\left(1\right)$. We provide proofs of the unimodularity of the cotangent bundle of a Lie group, and exploit this property to perform a mean/covariance propagation on the cotangent bundle of $Aff^{+}\left(1\right)$.

The mathematical apparatus developed can be applied to different PDEs in mathematical finance, such as those arising from stochastic volatility models or other forms of asset coupling in multi-asset models, and to linear convection-diffusion equations in transport theory, to name a few. Due to the unimodularity of the cotangent bundle group, the Lie group to which the derivatives are matched with need not be unimodular. Additionally, subsequent research can also be directed towards extending the cotangent bundle group construction presented here to groups with non-trivial centers, and to a general stability analysis of propagation schemes.

## Appendix A. Standard Solution of the Black-Scholes Equation

The one-asset Black-Scholes equation (9) can be solved exactly by first substituting $t^{\prime }=t$, and $V=u\left(a,t^{\prime }\right)e^{-rt^{\prime }}$ followed by $a=e^{x}$. These substitutions give us the following constant-coefficient one-dimensional diffusion equation:

(A1) $$\frac{\partial \tilde{u}}{\partial t^{\prime }}=\left(r-\frac{\sigma^{2}}{2}\right)\frac{\partial \tilde{u}}{\partial x}+\frac{\sigma^{2}}{2}\frac{\partial^{2}\tilde{u}}{\partial x^{2}},$$

where $u\left(a,t^{\prime }\right)=\tilde{u}\left(x,t^{\prime }\right)$. This form is consistent with the square-integrability condition used to derive the covariance propagation results in Section 4. Equation (A1) can be solved using the one-dimensional Fourier transform to yield the following solution,

(A2) $$\tilde{u}\left(x,t^{\prime }\right)=\tilde{u}\left(x,0\right)*\frac{1}{\sigma \sqrt{2\pi t^{\prime }}}\exp\left(\frac{-{\left(x-\left(\sigma^{2}/2-r\right)t^{\prime }\right)}^{2}}{2\sigma^{2}t^{\prime }}\right)$$

where ∗ is the one-dimensional convolution operator and u(a,0) is the initial condition. If the initial condition is a Dirac delta distribution centered at $a_{0}$ we have,

(A3) $$u_{f}\left(a,t^{\prime }\right)=\frac{1}{\sigma \sqrt{2\pi t^{\prime }}}\exp\left(-\frac{1}{2\sigma^{2}t^{\prime }}{\left[\log a-\log a_{0}+\left(r-\frac{\sigma^{2}}{2}\right)t^{\prime }\right]}^{2}\right),$$

which is the Green’s function for the one-asset Black-Scholes equation.

A similar procedure can be followed for the two-asset model (12) as well where we have $V=u\left(a,b,t^{\prime }\right)e^{-rt^{\prime }}$. Then substituting $a=e^{x}$ and $b=e^{y}$ we obtain for $\tilde{u}\left(x,y,t^{\prime }\right)=u\left(a,b,t^{\prime }\right)$,

(A4) $$\frac{\partial \tilde{u}}{\partial t^{\prime }}=\left(r-\frac{\sigma_{1}^{2}}{2}\right)\frac{\partial \tilde{u}}{\partial x}+\left(r-\frac{\sigma_{1}^{2}}{2}\right)\frac{\partial \tilde{u}}{\partial y}+\rho \sigma_{1}\sigma_{2}\frac{\partial^{2}\tilde{u}}{\partial x\partial y}+\frac{\sigma_{1}^{2}}{2}\frac{\partial^{2}\tilde{u}}{\partial x^{2}}+\frac{\sigma_{2}^{2}}{2}\frac{\partial^{2}\tilde{u}}{\partial y^{2}}.$$

The solution to Equation (A4) would be,

(A5) $$\tilde{u}\left(x,y,t^{\prime }\right)=\tilde{u}\left(x,y,0\right)*\frac{1}{2\pi \sigma_{1}\sigma_{2}\sqrt{1-\rho^{2}}t^{\prime }}\exp\left(-\frac{1}{2\sigma_{1}^{2}\sigma_{2}^{2}\left(1-\rho^{2}\right)t^{\prime }}\left(\sigma_{1}^{2}L_{2}^{2}+\sigma_{2}^{2}L_{1}^{2}-2\rho \sigma_{1}\sigma_{2}L_{1}L_{2}\right)\right),$$

where $L_{1}=x+\left(r-\sigma_{1}^{2}/2\right)t^{\prime }$ and $L_{2}=y+\left(r-\sigma_{2}^{2}/2\right)t^{\prime }$. Substituting x=loga and y=logb and using the initial condition $u\left(a,b,0\right)=\delta \left(a-a_{0}\right)\delta \left(b-b_{0}\right)$ we have,

(A6) $$u_{f}\left(a,b,t^{\prime }\right)=\frac{1}{2\pi \sigma_{1}\sigma_{2}\sqrt{1-\rho^{2}}t^{\prime }}\exp\left(-\frac{1}{2\sigma_{1}^{2}\sigma_{2}^{2}\left(1-\rho^{2}\right)t^{\prime }}\left(\sigma_{1}^{2}l_{2}^{2}+\sigma_{2}^{2}l_{1}^{2}-2\rho \sigma_{1}\sigma_{2}l_{1}l_{2}\right)\right),$$

where $l_{1}\left(t^{\prime }\right)=\log a+\left(r-\sigma_{1}^{2}/2\right)t^{\prime }-\log a_{0}$ and $l_{2}\left(t^{\prime }\right)=\log b+\left(r-\sigma_{2}^{2}/2\right)t^{\prime }-\log b_{0}$ where $u_{f}\left(a,b,t^{\prime }\right)$ is the Green’s function for the two-asset Black-Scholes equation.

## Appendix B. The Solution of a Diffusion Equation on a Unimodular Lie Group Is a Probability Density

Consider the diffusion equation for u(g,t) on an *N*-dimensional unimodular Lie group *G* of the form,

(A7) $$\frac{\partial u}{\partial t}=-\sum_{i=1}^{N}m_{i}\left(t\right)E_{i}^{R}u+\frac{1}{2}\sum_{i=1}^{N}\sum_{j=1}^{N}D_{ij}\left(t\right)E_{i}^{R}E_{j}^{R}u.$$

If we let $P=\int_{G}u\;dg$ and integrating both sides of (A7) with respect to the bi-invariant Haar measure dg, we have,

(A8) $$\frac{\partial P}{\partial t}=-\sum_{i=1}^{N}m_{i}\left(t\right)\int_{G}E_{i}^{R}u\;dg+\frac{1}{2}\sum_{i=1}^{N}\sum_{j=1}^{N}D_{ij}\left(t\right)\int_{G}E_{i}^{R}E_{j}^{R}u\;dg.$$

If *u* and $E_{i}^{R}u$ are ‘well-behaved’ in that their absolute values and the square of the functions integrate to a finite value over the group then, by integration by parts, both integrals on the right-hand-side vanish and we are left with ∂P/∂t=0 [17], which is the statement that the total probability (correct to a constant normalisation factor) is conserved. That is, if the initial condition u(g,0) is a probability density, u(g,t) will also be a probability density.

## Appendix C. Mean and Covariance in R N

For a probability density function $\tilde{u}\left(q,t\right)$ where $q\in R^{N}$, the mean μ(t) and covariance Σ(t) are defined as,

(A9) $$\int_{R^{N}}\left(q-\mu \left(t\right)\right)\tilde{u}\left(q,t\right)\;dq\;\dot{=}\;0,$$

(A10) $$\Sigma \left(t\right)\;\dot{=}\;\int_{R^{N}}\left(q-\mu \right)\;{\left(q-\mu \right)}^{T}\tilde{u}\left(q,t\right)\;dq.$$

If the probability density function was instead a function of g∈G, i.e., u(g,t) such that $\tilde{u}\left(q,t\right)=u\left(g\left(q\right),t\right)$, then a natural generalisation of the distance from the mean, (q−μ) in $R^{N}$, would be $\log(\mu^{-1}\circ g)$ in the Lie algebra, for μ∈G.

## Appendix D. Derivation of Equation (82)

The expression in (82) can be derived by first recognising that the matrix exponential of an N×N matrix *A* is given by the following infinite series,

(A11) $$\exp A=I_{N}+\sum_{n=1}^{\infty }\frac{A^{n}}{n!},$$

where $I_{N}$ is the identity matrix. The problem of evaluating $\log(\mu^{-1}\circ h)$ can be rewritten as finding the *A* such that $\exp A=\mu^{-1}\circ h$. Since *A* must be in the Lie algebra of the affine cotangent bundle group,

(A12) $$A=\left(\begin{array}{ccccc} 0 & \; & \alpha & \; & \gamma \\ 0 & \; & \beta & \; & \Gamma \\ 0 & \; & 0 & \; & 0 \end{array}\right).$$

Exponentiating (A12) using the series in (A11), we have,

(A13) $$\exp A=\left(\begin{array}{ccccc} 1 & \; & \alpha (1+\sum_{n=2}^{\infty }\frac{1}{n!}\beta^{n-1}) & \; & \gamma +\alpha \Gamma \sum_{n=2}^{\infty }\frac{1}{n!}\beta^{n-2}\\ 0 & \; & \exp(\beta ) & \; & \Gamma (1+\sum_{n=2}^{\infty }\frac{1}{n!}\beta^{n-1})\\ 0 & \; & 0 & \; & 0 \end{array}\right).$$

Matching expA with $\mu^{-1}\circ h$, where *h* is given by (68) and the general expression for μ is provided in (81), we obtain the following set of equations:

(A14) $$\exp\left(\beta \right)=a^{\prime }/a,$$

(A15) $$\alpha \left(1+\frac{1}{\beta }\sum_{n=2}^{\infty }\frac{\beta^{n}}{n!}\right)=\frac{b-b^{\prime }}{a},$$

(A16) $$\Gamma \left(1+\frac{1}{\beta }\sum_{n=2}^{\infty }\frac{\beta^{n}}{n!}\right)=a^{\prime }y,$$

(A17) $$\gamma +\frac{\alpha \Gamma }{\beta^{2}}\sum_{n=2}^{\infty }\frac{\beta^{n}}{n!}=x-b^{\prime }y.$$

These four equations can be solved for α,β,γ,Γ by using $\exp\left(\beta \right)=1+\sum_{n=1}^{\infty }(\beta^{n}/n!$). Then, applying the ∨ operator from (64) on (A12), one obtains (82).
